# Synthesis, antitumour activities and molecular docking of thiocarboxylic acid ester-based NSAID scaffolds: COX-2 inhibition and mechanistic studies

**DOI:** 10.1080/14756366.2018.1474878

**Published:** 2018-05-28

**Authors:** Adel S. El-Azab, Alaa A.-M. Abdel-Aziz, Laila A. Abou-Zeid, Walaa M. El-Husseiny, Ahmad M. El_Morsy, Manal A. El-Gendy, Magda A.-A. El-Sayed

**Affiliations:** a Department of Pharmaceutical Chemistry, College of Pharmacy, King Saud University, Riyadh, Saudi Arabia;; b Department of Organic Chemistry, Faculty of Pharmacy, Al-Azhar University, Cairo, Egypt;; c Department of Medicinal Chemistry, Faculty of Pharmacy, Mansoura University, Mansoura, Egypt;; d Department of Pharmaceutical Organic Chemistry, Faculty of Pharmacy, Mansoura University, Mansoura, Egypt;; e Department of Pharmaceutical Chemistry, Faculty of pharmacy, Horus university, New Damietta, Egypt

**Keywords:** NSAID thioesters, *in vitro*, antitumour, COX-1/COX-2, kinase inhibition assay, molecular docking

## Abstract

A new series of NSAID thioesters were synthesized and evaluated for their *in vitro* antitumor effects against a panel of four human tumor cell lines, namely: HepG2, MCF-7, HCT-116 and Caco-2, using the MTT assay. Compared to the reference drugs 5-FU, afatinib and celecoxib, compounds **2b**, **3b**, **6a**, **7a**, **7b** and **8a** showed potent broad-spectrum antitumor activity against the selected tumour cell lines. Accordingly, these compounds were selected for mechanistic studies about COX inhibition and kinase assays. *In vitro* COX-1/COX-2 enzyme inhibition assay results indicated that compounds **2b**, **3b**, **6a**, **7a**, **7b**, **8a** and **8 b** selectively inhibited the COX-2 enzyme (IC_50_ = ∼0.20–0.69 μM), with SI values of (>72.5–250) compared with celecoxib (IC_50_ = 0.16 μM, COX-2 SI: > 312.5); however, all the tested compounds did not inhibit the COX-1 enzyme (IC_50_ > 50 μM). On the other hand, EGFR, HER2, HER4 and cSrc kinase inhibition assays were evaluated at a 10 μM concentration. The selected candidates displayed limited activities against the various tested kinases; the compounds **2a**, **3b**, **6a**, **7a**, **7b** and **8a** showed no activity to weak activity (% inhibition = ∼0–10%). The molecular docking study revealed the importance of the thioester moiety for the interaction of the drugs with the amino acids in the active sites of COX-2. The aforementioned results indicated that thioester based on NSAID scaffolds derivatives may serve as new antitumor compounds.

## Introduction

Malignancy is global health problem and is the leading cause of death in children until fifteen years of age^1^. Non-steroidal anti-inflammatory drugs (NSAIDs) such as sulindac, indomethacin and celecoxib are commonly used for treating arthritis via inhibition of the cyclooxygenase enzyme (COX)^2^^,^^3^. COX-2 levels are over-expressed in human tumours, unlike in normal cells and could develop a tumorigenic potential^4^. Selective enzyme inhibition and restoration of normal apoptotic responses is known as COX-2-dependent anticancer mechanism^4–6^. On the other hand, COX-2-independent mechanisms function via apoptosis stimulation, angiogenesis arrest, or cancer cell growth inhibition by blocking signal transduction pathways for cell proliferation^7–10^.

**Figure 1.**
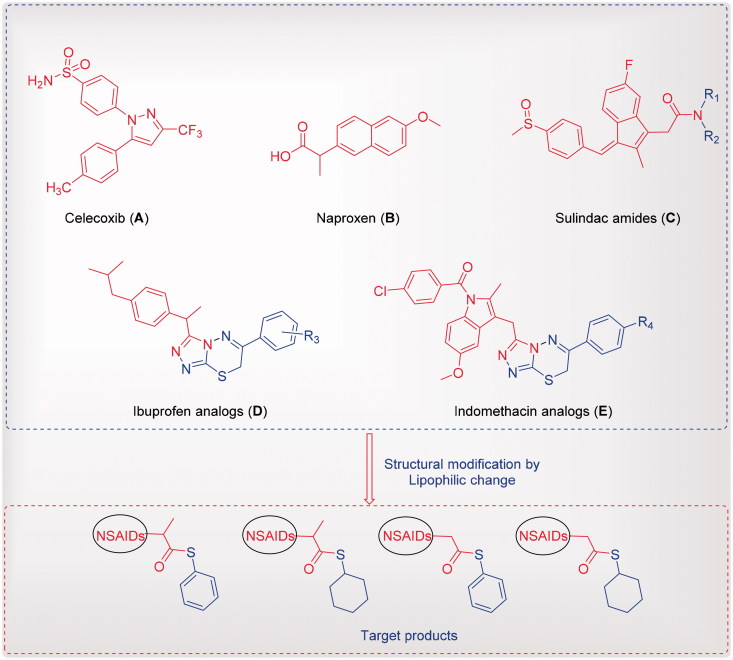
Reported NSAIDs and celecoxib as anticancer agents (**A**-**E**) and the designed compounds.

Drug repositioning development is a more important process for saving money and time than the production of a new drug^11^. NSAIDs and coxib such as naproxen, ibuprofen, indomethacin, sulindac, celecoxib and their analogues (Figure 1) have diverse scaffolds; modifying their basic structures is relatively safe, applicable for oral use, associated with multiple therapeutic features, such as analgesic, antipyretic, anti-inflammatory and anticancer activities^12–18^. For example, sulindac amides (Figure 1) showed a good activity against a panel of lymphoblastic leukemia cell lines in nanomolar concentrations^18^. Additionally, celecoxib reduced the number and size of colorectal polyps in adenomatous polyposis (Figure 1)^19–21^. Antiproliferative and apoptosis effects of celecoxib in colon, stomach, lung, prostate and pancreatic cancer cells have been observed by selective COX-2 inhibition^22–25^. On the other hand, a combination of drugs (NSAIDs) such as indomethacin, sulindac, tolmetin, acemetacin, zomepirac and mefenamic acid at non-toxic levels, and different chemotherapeutic drugs such as anthracyclines (doxorubicin, daunorubicin and epirubicin), in addition to VP-16, vincristine and teniposide, led to a significantly synergistic cytotoxicity of these chemotherapeutic drugs in the human COR L23R, DLKP, A549 and COR L23P lung cancer cell lines, and the human HL60/ADR leukaemia cell line^3^.

Continuing our studies as an attempt to develop effective cytotoxic agents^26–42^, we synthesised some NSAIDs conjugated to thioester moieties and evaluated their cytotoxic activities. Additionally, *in vitro* COX-1/COX-2 enzyme and kinase inhibitory assays were investigated for the most active compounds, to identify their mode of action.

A molecular docking technique was used in order to predict the binding geometry requirements of the target molecules, which is important for the antitumour activity.

## Experimental

Melting points were recorded on a Barnstead 9100 Electrothermal melting apparatus. IR spectra (KBr) were recorded on an FT-IR Perkin-Elmer spectrometer (ν cm^−1^). ^1^H and ^13^C NMR spectra were recorded on Bruker 500 or 700 MHz spectrometers using DMSO-d_6_ or CDCl_3_ as the solvent. Microanalytical data (C, H and N) were obtained using a Perkin-Elmer 240 analyser and the proposed structures were within ±0.4% of the theoretical values. Mass spectra were recorded on a Varian TQ 320 GC/MS/MS mass spectrometer. NSAIDs thioester was obtained according to reported method^43^.

## General method for the preparation of NSAIDs thioester

Trifluoroacetic acid (0.5 mmol) was added dropwise to a mixture of NSAIDs (0.1 mmol) and thiol (0.5 mmol) in dry acetonitrile that was heated for 10–12 h at 60 °C. The reaction mixture was cooled, quenched using ammonium chloride solution, extracted with ethylacetate, washed with brine and dried over anhydrous sodium sulphate; the solvent was then evaporated, and the product obtained was chromatographed with hexane and CHCl_3_.

### S-phenyl-2–(4-isobutylphenyl)propanethioate (1a)^44^

Yield, 89%; colourless oil; IR (KBr) ν_max_/cm^−1^ 1700.69 (CO), 738.10, 690.48 (CS); ^1^H NMR (500 MHz, CDCl_3_): *δ* 7.47–7.51 (*m*, 5H), 7.44 (d, 2H, *J* = 8.0 Hz), 7.30 (d, 2H, *J* = 7.5 Hz), 4.13 (d, 1H, *J* = 7.0 Hz), 2.64 (d, 2H, *J* = 7.0 Hz), 2.04 (*t*, 1H, *J* = 6.5 Hz), 1.73 (d, 3H, *J* = 7.0 Hz), 1.08 (d, 6H, *J* = 6.5); ^13 ^C NMR (125 MHz, CDCl_3_): δ 18.8, 22.6, 30.3, 45.2, 53.9, 127.3, 127.6, 127.9, 128.2, 129.2, 129.3, 129.6, 134.6, 136.6, 141.1, 199.1; MS; *m/z* (298).

### S-cyclohexyl-2–(4-isobutylphenyl)propanethioate (1b)

Yield, 81%; colourless oil; ^1^H NMR (500 MHz, CDCl_3_): *δ* 7.27 (d, 2H, *J* = 8.0 H), 7.15 (d, 2H, *J* = 8.0 H), 3.88 (d, 1H, *J* = 7.0 Hz), 3.52 (*s*, 1H), 2.52 (d, 2H, *J* = 7.0 Hz), 1.98 (*s*, 1H), 1.90–1.94 (*q*, 2H, *J* = 7.0, 6.5 Hz), 1.74 (*s*, 1H), 1.69 (*s*, 1H), 1.62 (d, 1H, *J* = 12 Hz), 1.56 (d, 3H, *J* = 7.0 Hz), 1.35–1.45 (*m*, 4H), 1.29 (*s*, 1H), 0.96 (d, 6H, *J* = 6.5); ^13 ^C NMR (125 MHz, CDCl_3_): δ 18.6, 22.4, 25.6, 26.0, 30.2, 32.9, 33.1, 42.4, 45.1, 54.0, 127.6, 129.3, 137.3, 140.6, 200.9; MS; *m/z* (304).

### S-phenyl-2–(3-benzoylphenyl)propanethioate (2a)

Yield, 88%; mp: 96–98 °C; IR (KBr) ν_max_/cm^−1^ 1668.97 (CO), 746.66, 694.49 (CS); ^1^H NMR (500 MHz, CDCl_3_): *δ* 7.73 (d, 3H, *J* = 8.5 Hz), 7.63 (d, 1H, *J* = 7.5 Hz), 7.50 (*t*, 2H, *J* = 6.0 Hz), 7.39 (dd, 3H, *J* = 7.5, 11.5 HZ) 7.28 (*s*, 5H), 3.99 (*q*, 1H, *J* = 6.5, 7.0 Hz), 1.52 (d, 3H, *J* = 7.0 Hz); ^13 ^C NMR (125 MHz, CDCl_3_): δ 16.3, 51.5, 125.3, 126.0, 126.4, 126.9, 127.1, 127.4, 127.8, 129.6, 130.2, 132.1, 135.1, 135.7, 137.5, 194.0, 196.3; MS *m/z* (346).

### S-cyclohexyl-2–(3-benzoylphenyl)propanethioate (2b)

Yield, 81%; mp: 69–70 °C; ^1^H NMR (500 MHz, CDCl_3_): *δ* 7.81 (d, 2H, *J* = 7.0 Hz), 7.76 (s, 1H), 7.70 (d, 1H, *J* = 7.5 Hz), 7.55–7.60 (*m*, 2H), 7.43–7.49 (*m*, 3H), 3.93 (*q*, 1H, *J* = 7.0 & 6.5 Hz), 3.48 (*s*, 1H), 1.91 (*s*, 1H), 1.84 (d, 1H, *J* = 10 Hz), 1.67 (d, 2H, *J* = 14.0 Hz), 1.55 (d, 4H, *J* = 7.0 Hz), 1.34–1.40 (*m*, 4H), 1.25 (*s*, 1H); ^13 ^C NMR (125 MHz, CDCl_3_): δ 18.4, 25.5, 25.9, 32.8, 32.9, 42.6, 54.0, 128.3, 128.6, 129.1, 129.7, 130.1, 131.8, 132.5, 137.4, 137.8, 140.3, 196.3, 200.4; MS *m/z* (352).

### S-phenyl-2–(2-fluoro-[1,1′-biphenyl]-4-yl)propanethioate (3a)

Yield, 90%; mp: 85–86 °C; IR (KBr) ν_max_/cm^−1^ 1694.14 (CO), 736.75, 687.25 (CS); ^1^H NMR (500 MHz, CDCl_3_): *δ* 1.52 (d, 3H, *J* = 7 Hz), 3.94 (dd, 1H, *J* = 6.5 & 7.0 Hz), 7.46 (d, 2H, J = 12.5 Hz), 7.29–7.41 (*m*, 9H), 7.05–7.14 (*m*, 2H); ^13 ^C NMR (125 MHz, CDCl_3_): δ 18.6, 53.4, 115.5, 115.7, 124.0, 127.5, 127.7, 128.5, 129.0, 129.2, 129.4, 130.9, 131.0, 134.4, 135.4, 140.7, 140.8, 158.7, 160.7, 198.5; MS; *m*/*z* (336).

### S-cyclohexyl-2–(2-fluoro-[1,1′-biphenyl]-4-yl)propanethioate (3b)

Yield, 80%; mp: 90–92 °C; IR (KBr) ν_max_/cm^−1^ 1672.76 (CO), 751.18, 690.19 (CS); ^1^H NMR (500 MHz, CDCl_3_): *δ* 7.45 (d, 2H, *J* = 7.5 Hz), 7.25–7.36 (*m*, 4H), 7.03–7.07 (*m*, 2H,), 3.79 (t, 1H, *J* = 7.0 Hz), 3.40 (*s*, 1H), 1.76–1.84 (*m*, 2H), 1.57–1.60 (d, 2H, *J* = 13 Hz), 1.45 (d, 4H, *J* = 7.0 H), 1.23–1.33 (*m*, 4H), 1.17 (*s*, 1H); ^13 ^C NMR (125 MHz, CDCl_3_): δ 18.4, 25.5, 25.9, 32.8, 33.0, 42.7, 53.6, 115.4, 115.6, 123.8, 123.9, 127.6, 127.9, 128.0, 128.4, 128.9, 130.7, 130.8, 135.5, 141.3, 141.4, 158.7, 160.6, 200.5; MS *m*/*z* (342).

### 2-[(Phenylthio)carbonyl]phenyl acetate (4a)^45^

Yield, 84%; mp: 72–73 °C; ^1^H NMR (500 MHz, CDCl_3_): *δ* 7.91 (dd, 1H, *J* = 1.0 Hz), 7.42–7.45 (*m*, 6H) 6.87–6.92 (*m*, 2H), 2.24 (*s*, 3H); ^13 ^C NMR (125 MHz, CDCl_3_): *δ* 29.7, 118.36, 119.4, 126.0, 128.9, 129.4, 130.0, 135.5, 136.3, 159.7, 195.8. MS *m/z* (272).

### 2-[(Cyclohexylthio)carbonyl]phenyl acetate (4b)

Yield, 80%; mp: 55–56 °C; ^1^H NMR (500 MHz, CDCl_3_): *δ* 7.78 (d, 1H, *J* = 8.0 Hz), 7.35 (t, 1H, *J* = 7.0 Hz), 6.88 (d, 1H, *J* = 8.0 Hz), 6.79 (*t*, 1H, *J* = 7.0 Hz), 3.66 (*s*, 1H), 2.26 (*s*, 2H), 1.95 (d, 2H, *J* = 10 Hz), 1.69 (*t*, 2H, *J* = 4.5 Hz), 1.56 (d, 1H, *J* = 8.5 Hz), 1.38–1.49 (*m*, 4H) 1.25 (d, 2H, *J* = 8.5 Hz); ^13 ^C NMR (125 MHz, CDCl_3_): *δ* 25.5, 25.9, 29.7, 33.01, 42.5, 118.1, 119.1, 120.2, 128.8, 135.6, 159.5, 197.4; MS *m*/*z* (278).

### S-phenyl-(S)-2–(6-methoxynaphthalen-2-yl)propanethioate (5a)^44^

Yield, 88%; mp: 115–117 °C; IR (KBr) ν_max_/cm^−1^ 1694.16 (CO), 738.16, 683.87 (CS); ^1^H NMR (500 MHz, CDCl_3_): *δ* 7.88 (*s*, 4H), 7.59 (d, 3H, *J* = 7.5 Hz), 7.49 (*s*, 3H), 7.31(d, 1H, *J* = 8.5 Hz), 4.28 (d, 1H, *J* = 6.5 Hz), 4.05 (*s*, 3H), 1.80 (d, 3H, *J* = 6.0 Hz); ^13 ^C NMR (125 MHz, CDCl_3_): δ 18.7, 54.1, 55.3 105.7, 119.1, 126.4, 126.9, 127.4, 128.0, 129.0, 129.1, 129.3, 129.4, 134.0, 134.5, 134.7, 157.8, 199.2; MS *m*/*z* (322).

### S-cyclohexyl-(S)-2–(6-methoxynaphthalen-2-yl)propanethioate (5b)

Yield, 84%; mp: 105–106 °C; IR (KBr) ν_max_/cm^−1^ 1679.27 (CO), 741.06, 688.41 (CS); ^1^H NMR (500 MHz, CDCl_3_): *δ* 7.59–7.64 (*m*, 3H), 7.30 (dd, 1H, *J* = 1.0 Hz), 7.05 (dd, 1H, *J* = 2.0 Hz), 7.03 (*s*, 1H), 3.88 (d, 1H, *J* = 7.0 Hz), 3.82 (*s*, 3H), 3.37 (*s*, 1H), 1.82 (d, 1H, *J* = 8.5 Hz), 1.72 (*t*, 1H, *J* = 5.0 & 5.5 Hz), 1.59–1.46 (*m*, 6H), 1.30–1.13 (*m*, 5H); ^13 ^C NMR (125 MHz, CDCl_3_): δ 18.5, 25.5, 26.0, 32.9, 33.0, 42.5, 54.2, 55.3, 105.6, 119.0, 126.4, 126.5, 127.1, 128.9, 129.3, 133.8, 135.3, 157.7, 201.2; MS *m*/*z* (328).

### S-phenyl-2–(2-((2,6-dichlorophenyl)amino)phenyl)ethanethioate (6a)

Yield, 86%; mp: 101–102 °C; IR (KBr) ν_max_/cm^−1^ 1679.27 (CO), 741.06, 688.41 (CS); ^1^H NMR (500 MHz, CDCl_3_): *δ* 10.01 (*s*, 1H), 7.40–7.42 (d, 2H, *J* = 8.0 Hz), 7.24–7.29 (*m*, 4H), 7.09–7.12 (*t*, 3H, *J* = 7.5 Hz), 6.99–7.00 (d, 2H, *J* = 7.5 Hz), 6.30–6.32 (d, 1H, *J* = 7.5 Hz), 3.68 (s, 2H); ^13 ^C NMR (125 MHz, CDCl_3_): δ 35.7, 109.1, 123.0, 124.3, 124.8, 125.2, 127.7, 127.9, 129.0, 130.4, 130.8, 131.0, 134.5, 135.5, 139.6, 143.3, 198.0; MS *m/z* (388).

### S-cyclohexyl-2–(2-((2,6-dichlorophenyl)amino)phenyl)ethanethioate (6b)

Yield, 83%; mp: 88–90 °C; ^1^H NMR (500 MHz, CDCl_3_): *δ* 7.38–7.40 (d, 2H, *J* = 8.0 Hz) 7.18–7.24 (*m*, 2H), 7.08–7.11 (*t*, 1H, *J* = 7.5 Hz), 6.97–7.00 (*t*, 1H, *J* = 7.5 Hz), 6.29–6.31 (d, 1H, *J* = 8.0 Hz), 3.70 (*s*, 2H), 3.01 (*s*, 1H), 2.01–2.00 (d, 4H, *J* = 9.0 Hz), 1.69–1.67 (*t*, 2H, *J* = 8.5 Hz), 1.49–1.47 (*t*, 2H, *J* = 8.5 Hz), 1.37–1.36 (d, 2H, *J* = 8.5 Hz); ^13 ^C NMR (125 MHz, CDCl_3_): δ 22.7, 29.7, 35.7, 39.2, 41.0, 123.1, 124.3, 124.8, 127.9, 129.0, 129.5, 130.4, 130.8, 131.0, 135.5, 143.3, 198.0; MS *m*/*z* (394).

### S-phenyl-2–(1-(4-chlorobenzoyl)-5-methoxy-2-methyl-1H-indol-3-yl)ethanethioate (7a)^46^

Yield, 86%; mp: 133–135 °C; IR (KBr) ν_max_/cm^−1^ 1671.45, 1604.72 (CO), 745.04, 693.51 (CS); ^1^H NMR (500 MHz, CDCl_3_): *δ* 7.59 (d, 2H, *J* = 8.5 Hz), 7.38 (d, 2H, *J* = 8.5 Hz), 7.29 (s, 5H), 6.92 (d, 1H, *J* = 2.0 Hz), 6.82 (d, 1H, *J* = 9.0 Hz), 6.61 (dd, 1H, *J* = 2.0, 9.0 Hz), 3.87 (*s*, 2H), 3.76 (*s*, 3H), 2.36 (*s*, 3H); ^13 ^C NMR (125 MHz, CDCl_3_): δ 13.5, 39.1, 55.7, 101.2, 111.8, 127.6, 129.1, 129.2, 129.4, 130.5, 130.9, 131.2, 133.7, 134.4, 136.8, 139.4, 156.2, 168.3, 195.1; MS *m*/*z* (449), (M + 2, 451).

### S-cyclohexyl-2–(1-(4-chlorobenzoyl)-5-methoxy-2-methyl-1H-indol-3-yl)ethanethioate (7b)^46^

Yield, 83%; mp: 97–98 °C; IR (KBr) ν_max_/cm^−1^ 1672.24, 1600.15 (CO), 830.24, 749.96 (CS); ^1^H NMR (700 MHz, DMSO-d_6_): *δ* 7.76 (d, 2H, *J* = 5.5 Hz), 7.64 (d, 2H, *J* = 5.5 Hz), 7.05 (s, 1H), 6.93 (d, 1H, *J* = 6.5 Hz), 7.72 (d, 1H, *J* = 6.5 Hz), 3.98 (*s*, 2H), 3.75 (*s*, 3H), 2.55–2.51 (m, 1H), 2.23 (*s*, 3H), 1.79 (*s*, 2H), 1.59 (*s*, 2H), 1.50 (d, 1H, *J* = 8.5 Hz), 1.33–1.30 (*m*, 4H), 1.20 (*s*, 1H); ^13 ^C NMR (125 MHz, CDCl_3_): δ 13.8, 25.4, 25.8, 32.8, 40.2, 42.4, 55.8, 102.2, 111.9, 112.8, 115.0, 129.5, 130.6, 130.9, 131.6, 134.4, 136.5, 138.2, 156.0, 168.3, 196.8; MS *m*/*z* (456), (M + 2, 458).

### S-phenyl-2–(5-fluoro-2-methyl-1–(4-(methylsulfinyl)benzylidene)-1H-inden-3-yl)ethanethioate (8a)

Yield, 78%; mp: 66–68 °C; IR (KBr) ν_max_/cm^−1^ 1700.49 (CO), 1021 (SO), 734.05, 684.77 (CS); ^1^H NMR (500 MHz, CDCl_3_): *δ* 7.02–7.47 (*m*, 13H), 3.93–3.94 (d, 2H, *J* = 7.0 Hz), 2.27 (s, 3H), 1.46–1.52 (dd, 3H, *J* = 7.0 Hz); ^13 ^C NMR (125 MHz, CDCl_3_): *δ* 18.6, 21.3, 53.3, 115.5, 115.7, 124.0, 127.7, 128.5, 129.0, 129.3, 130.0, 130.9, 134.4, 135.4, 139.7, 140.9, 158.7, 160.7, 198.9; MS *m*/*z* (448).

### S-cyclohexyl-2–(5-fluoro-2-methyl-1–(4-(methylsulfinyl)benzylidene)-1H-inden-3-yl)ethanethioate (8b)

Yield, 75%; mp: 121–122 °C; IR (KBr) ν_max_/cm^−1^ 1692.84 (CO), 859.17, 808.66 (CS), (SO); ^1^H NMR (700 MHz, DMSO-d_6_): *δ* 7.67–7.63 (*m*, 5H, 7.05 (*s*, 1H), 6.93 (d, 1H, *J* = 9.1 Hz), 6.72 (d, 1H, *J* = 9.1 Hz), 3.98 (*s*, 2H), 3.75 (*s*, 3H), 2.23 (*s*, 3H), 1.799 (*s*, 2H), 1.59 (*s*, 2H), 1.50 (d, 1H, *J* = 11.9 Hz), 1.31 (*t*, 4H, *J* = 10.5 & 9.8 Hz), 1.20 (*s*, 1H); ^13 ^C NMR (176 MHz, DMSO-d_6_): *δ* 13.8, 25.4, 25.8, 32.8, 39.3, 42.4, 55.8, 102.0, 111.9, 112.8, 115.0, 129.5, 130.6, 130.9, 131.6, 134.4, 136.5, 138.2, 156.0, 168.3, 196.8; MS *m*/*z* (454).

## Biological testing

### Antitumor evaluation

The evaluation of the antitumour activity was performed using tetrazolium salt MTT (3–(4,5-dimethyl-2-thiazolyl)-2,5-diphenyl-2*H*-tetrazolium bromide) assay as reported^47–50^.

### *In vitro* cyclooxygenase (COX) inhibition assay

The colorimetric COX (ovine) inhibitor screening assay kit (kit catalogue number 560101, Cayman Chemical, Ann Arbor, MI) was utilized according to the manufacturer’s instructions to examine the ability of the test compounds and the reference drugs to inhibit the COX-1/COX-2 isozymes^51^^,^^52^.

### Kinase inhibition assay

The assay for Kinases was performed at BPS Bioscience Inc. 6044 Cornerstone Court West, Ste. E, San Diego, CA 92121, USA using Kinase-Glo Plus luminescence kinase assay kit (Promega). Luminescence signal was measured using a BioTek *Synergy 2* microplate reader^53^.

### Docking methodology

All modelling experiments were conducted with MOE programs running on PC computer [MOE 2008.10 of Chemical Computing Group. Inc]^54^. The docking protocol is summarized in supporting information^51^^,^^52^^,^^55–57^.

## Results and discussion

### Chemistry

**Scheme 1.**
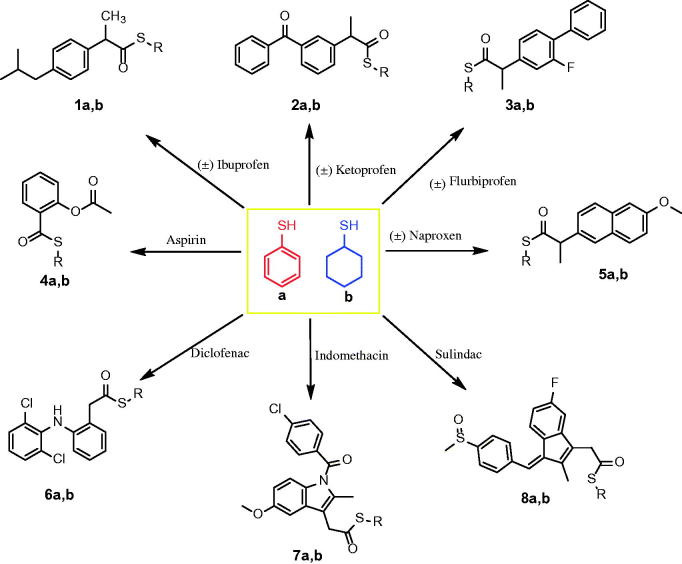
Synthesis of the designed thiocarboxylic acid esters of NSAIDs.

The new thioesters were synthesized by the reaction of the carboxylic acid group of NSAIDs with thiophenol and cycloxanethiol in the presence of trifluoroacetic acid (TFA)^43^. The newly synthesized thioesters (Scheme 1) were confirmed by the presence of the carbonyl group (C=O) at 1700–1669 cm^−1^ and stretching of the (C–S) group at 859–683 cm^−1^ in the IR spectra. Additionally, the newly synthesised thioesters were confirmed by a characteristic peak at 201.21–195.15 ppm attributable to the (S–C=O) group in addition to the characteristic peaks of the cyclohexane moiety at 25.40–42.42 ppm or aromatic peaks of the thiophenol moiety in the aromatic region of the ^13^C NMR spectra. The ^1^H NMR spectra of the new thioesters showed a singlet peak because of the S–CH moiety of S-cyclohexane at 3.66–3.37 ppm, as well as the other 10 protons of the cyclohexane moiety in the aliphatic region or the aromatic peaks of the thiophenol moiety in the aromatic region.

### Biological evaluation

#### Antitumor evaluation using MTT assay

The synthesised thioesters were evaluated for their *in vitro* antitumor effects using the standard 3–(4,5-dimethylthiazol-2-yl)-2,5-diphenyltetrazolium bromide (MTT) method^47–50^ against a panel of four human tumour cell lines: hepatocellular carcinoma cell line (HepG2), breast cancer cell line (MCF-7), colon cancer cell line (HCT-116) and colorectal cancer cell line (Caco-2). The antitumor activities of the newly synthesized compounds **1a,b**–**8a,b** compared with those of the reference drugs, 5-FU, afatinib and celecoxib are shown in Table 1. In the present study, the active compounds revealed a characteristic selectivity potential, in addition being broad-spectrum compounds. With respect to the selectivity against the hepatocellular carcinoma cell line (HepG2), the thioesters **2b, 3b, 5b**, **7a**,**b** and **8a** showed potent antitumour activity with IC_50_ values of 7.35–19.74 μM, while thioesters **5a**, **6a** and **8b** showed moderate antitumour activity against HepG2 cell line, with IC_50_ values 22.30–36.75 μM, compared to the reference drugs 5-FU, afatinib and celecoxib (IC_50_ = 7.91, 5.40 and 25.60 μM, respectively). Additionally, the MCF-7 cell line showed a high sensitivity to the thioesters **2a**, **3b, 6b**, **7a**,**b** and **8a**, with IC_50_ values of 6.11–17.10 μM, whereas the thioesters **4b**, **5a**,**b** and **8b** showed a moderate antitumour activity, with IC_50_ values 28.90–46.52 μM, compared with the reference drugs 5-FU, afatinib and celecoxib (IC_50_ = 5.43, 7.1 and 31.28 μM, respectively). Moreover, the colon cancer cell line (HCT-116) had a strong susceptibility to the thioesters **2a**, **7a** and **8a** with IC_50_ values of 9.73–18.71 μM, but a moderate susceptibility to the thioesters **3b**, **5b**, **6a**, **7b** and **8b**, with IC_50_ values of 23.76–46.92 μM compared to the reference drugs, 5-FU, afatinib and celecoxib (IC_50_ = 5.32, 6.20 and 29.54 μM, respectively). Additionally, the thioesters **2b, 6a, 7a** and **8a** exhibited a strong antitumour activity against colorectal cancer cell line (Caco-2), with IC_50_ values of 10.16–21.73 μM, whereas the thioesters **5b**, **6b**, **8b** and **9b** showed a moderate antitumour activity, with IC_50_ values of 26.81–43.79 μM, when compared to the reference drugs 5-FU, afatinib and celecoxib (IC_50_ = 6.85, 7.70 and 42.74 μM, respectively).

**Table 1.** *In vitro* antitumor activity of 5-fluorouracil, afatinib, celecoxib, and the tested compounds.

Compd No.	IC_50_ (μM)^a^			
	HepG2^b^	MCF-7^c^	HCT-116^d^	Caco-2^e^
5-FU	7.91 ± 0.28	5.43 ± 0.20	5.32 ± 0.17	6.85 ± 0.34
Afatinib	5.4 ± 0.25	7.1 ± 0.49	6.2 ± 0.67	7.7 ± 0.57
Celecoxib	25.6 ± 2.3	31.28 ± 2.5	29.54 ± 2.1	42.74 ± 3.1
**1a**	85.12 ± 4.53	80.41 ± 4.58	89.63 ± 4.68	94.83 ± 4.92
**1b**	>100	>100	97.56 ± 5.12	>100
**2a**	59.83 ± 3.55	48.11 ± 3.15	61.29 ± 3.97	72.19 ± 4.06
**2b**	9.36 ± 0.79	11.86 ± 1.13	18.71 ± 1.50	21.73 ± 1.90
**3a**	68.75 ± 3.87	63.61 ± 3.62	78.11 ± 4.08	76.52 ± 4.38
**3b**	10.52 ± 0.98	13.73 ± 1.19	23.76 ± 1.80	26.81 ± 2.17
**4a**	71.08 ± 4.11	73.65 ± 3.92	85.40 ± 4.57	80.20 ± 4.50
**4b**	63.62 ± 3.91	46.52 ± 2.84	76.54 ± 4.22	68.75 ± 3.79
**5a**	36.75 ± 2.70	42.61 ± 2.67	51.17 ± 3.71	63.78 ± 3.58
**5b**	19.74 ± 1.57	28.90 ± 1.58	39.52 ± 2.61	35.60 ± 2.62
**6a**	26.76 ± 2.08	6.11 ± 0.31	46.92 ± 3.23	10.16 ± 0.92
**6b**	>100	95.26 ± 4.96	91.22 ± 4.96	>100
**7a**	7.86 ± 0.39	9.65 ± 0.96	14.58 ± 1.24	18.13 ± 1.73
**7b**	14.91 ± 1.38	17.10 ± 1.40	34.05 ± 2.25	29.14 ± 2.45
**8a**	7.35 ± 0.34	8.62 ± 0.72	9.73 ± 0.85	15.44 ± 1.37
**8b**	22.30 ± 1.96	34.09 ± 2.07	46.71 ± 2.93	43.79 ± 2.96

^a^IC_50_, compound concentration required to inhibit tumour cell proliferation by 50% (mean ± SD), *n* = 3.

^b^Human hepato-cellular carcinoma cell line (HepG2).

^c^Human breast adenocarcinoma cell line (MCF-7).

^d^Human colon cancer cell line (HCT-116).

^e^Human colorectal cancer cell line (Caco-2).

IC_50_, (μM): 1–10 (very strong), 11–25 (strong), 26–50 (moderate), 51–100 (weak), above 100 (non-cytotoxic).

5-FU: 5-Fluorouracil.

The thioesters **3b** and **7b** displayed a broad-spectrum antitumor activity against the HepG2 cell line (IC_50_ = 10.52 and 14.91 μM respectively) and MCF-7 cell line (IC_50_ = 13.73 and 17.10 μM respectively), while the thioester **6a** showed a broad-spectrum antitumor activity against the MCF-7 cell line and Caco-2 cell line (IC_50_ = 6.11 and 10.16 μM, respectively). Additionally, the thioesters **2b**, **7a** and **8a** showed strong antitumour activities against the HepG2 cell line (IC_50_ = 9.36, 7.86 and 7.35 μM, respectively), MCF-7 cell line (IC_50_ = 11.86, 9.65 and 8.62 μM respectively), HCT-116 cell line (IC_50_ = 18.71, 14.58 and 9.73 μM, respectively), and Caco-2 cell line (IC_50_ = 21.73, 18.13 and 15.44 μM, respectively). On the other hand, the thioesters **1a**, **b**, **2b**, **3a**, **4a** and **6b** showed a weak antitumor activity with IC_50_ values from 48.11 to >100 μM.

#### *In vitro* COX inhibition assay

Compounds that showed promising and potent antitumor activities (Table 1) were subjected to *in vitro* COX-1/COX-2 inhibition assays. As indicated in Table 2, seven compounds were selected for *in vitro* COX-1/COX-2 evaluation (Table 2). IC_50_ (represented in μM) (The half-maximal inhibitor concentration) values were determined^56–59^, and the SI values were calculated^51^^,^^52^^,^^55^ as IC_50_ (COX-1)/IC_50_ (COX-2). Interestingly, some of the tested compounds selectively inhibited COX-2 (IC50 = 0.20–0.69 μM); however, all the tested compounds did not inhibit COX-1 (IC_50_ > 50 μM).

**Table 2.** *In vitro* COX-1/COX-2 enzyme inhibition assay.

Compd No.	IC50 (μM)^a^		SI^b^
	COX-1	COX-2	
Celecoxib	>50	0.16 ± 0.011	>312.5
**2b**	>50	0.66 ± 0.052	>75.8
**3b**	>50	0.69 ± 0.057	>72.5
**6a**	>50	0.25 ± 0.017	>200.0
**7a**	>50	0.22 ± 0.019	>227.3
**7b**	>50	0.49 ± 0.044	>102.0
**8a**	>50	0.20 ± 0.016	>250.0
**8b**	>50	0.60 ± 0.055	>83.3

**^a^**IC_50_ value is the compound concentration required to produce a 50% inhibition of COX-1 or COX-2, calculated as the mean of two determinations using the ovine COX-1/COX-2 assay kit (catalog no. 560101, Cayman Chemicals Inc., USA); the deviation from the mean is <10% of the mean value.

**^b^**Selectivity index (COX-1 IC_50_/COX-2 IC_50_).

Compounds **2b**, **3b**, **7b** and **8b** possessed good COX-2 inhibitory activity with IC_50_ values of 0.66, 0.69, 0.49 and 0.60 μM, and SI values of >75.8, 72.5, 102.0 and 83.3, respectively, comparable to that of celecoxib (IC_50_ = 0.16 μM, COX-2 SI: > 312.5). Furthermore, compounds **6a**, **7a** and **8a** showed a potent selective inhibition of COX-2, with IC_50_ values of 0.25, 0.22 and 0.20 μM, and SI values of >200, 227 and 250, respectively, compared to those of celecoxib (IC_50_ = 0.16 μM, COX-2 SI: > 312.5). The structure–activity relationships of the COX inhibition assays revealed the following: (i) substituted thiophenyl derivatives, such as compounds **6a**, (IC_50_ = 0.25 μM, COX-2 SI: >200), **7a** (IC_50_ = 0.22 μM, COX-2 SI: >227) and **8a** (IC50 = 0.20 μM, COX-2 SI: >250), were more effective COX-2 inhibitors than substituted thiocyclohexyl derivatives, such as compounds **2b** (IC_50_ = 0.66, μM, COX-2 SI: >75.8), **3b** (IC_50_ = 0.69 μM, COX-2 SI: >72.5), **7b** (IC_50_ = 0.49 μM, COX-2 SI: >102.0) and **8b** (IC_50_ = 0.60 μM, COX-2 SI: >83.3); (ii) S-phenyl-2–(5-fluoro-2-methyl-1–(4-(methylsulfinyl)benzylidene)-1*H*-inden-3-yl)ethanethioate (**8a)** was more effective than S-phenyl-2–(1-(4-chlorobenzoyl)-5-methoxy-2-methyl-1*H*-indol-3-yl)ethanethioate (**7a**) and S-phenyl 2–(2-((2,6-dichlorophenyl)amino)phenyl)ethanethioate (**6a**), while the latter was less effective than compound **7a**; (iii) the substituted thiocyclohexyl derivative, S-cyclohexyl 2–(1-(4-chlorobenzoyl)-5-methoxy-2-methyl-1*H*-indol-3-yl)ethanethioate (**7b**) was more effective than S-cyclohexyl-2–(5-fluoro-2-methyl-1–(4-(methylsulfinyl)benzylidene)-1*H*-inden-3-yl)ethanethioate (**8b**), S-cyclohexyl 2–(3-benzoylphenyl)propanethioate (**2b**) and S-cyclohexyl 2–(4-isobutylphenyl)propanethioate (**3b**).

#### *In vitro* kinase assay

Accordance to the cytotoxicity activity of the newly synthesized compounds (Table 1), six compounds were selected for further mechanistic investigations about the kinases, EGFR, HER2, HER4 and cSrc. The results of kinase inhibition assays indicated that compounds **2a**, **3b**, **6a**, **7a**, **7b** and **8a** showed limited activities against the kinase enzymes. As shown in Table 3, all the compounds showed no or weak activities against HER2, HER4 and cSrc, as indicated by their % inhibition when used at a concentration of 10 μM (% inhibition = ∼0–10%), comparable to the 81–100% inhibition of the reference drug staurosporine, used at a concentration of 1 μM (Table 3).

**Table 3.** % inhibitory effect of the compounds on kinase activities.

Compd No.	% inhibition of 10 μM			
	EGFR	HER2	HER4	cSrc
Staurosporine^a^	94	81	100	100
**2b**	0	1	5	4
**3b**	0	0	3	3
**6a**	6	1	5	5
**7a**	0	0	4	1
**7b**	1	0	3	3
**8a**	6	0	10	4

^a^Staurosporine used in 1 μM concentration.

## Docking studies

**Figure 2.**
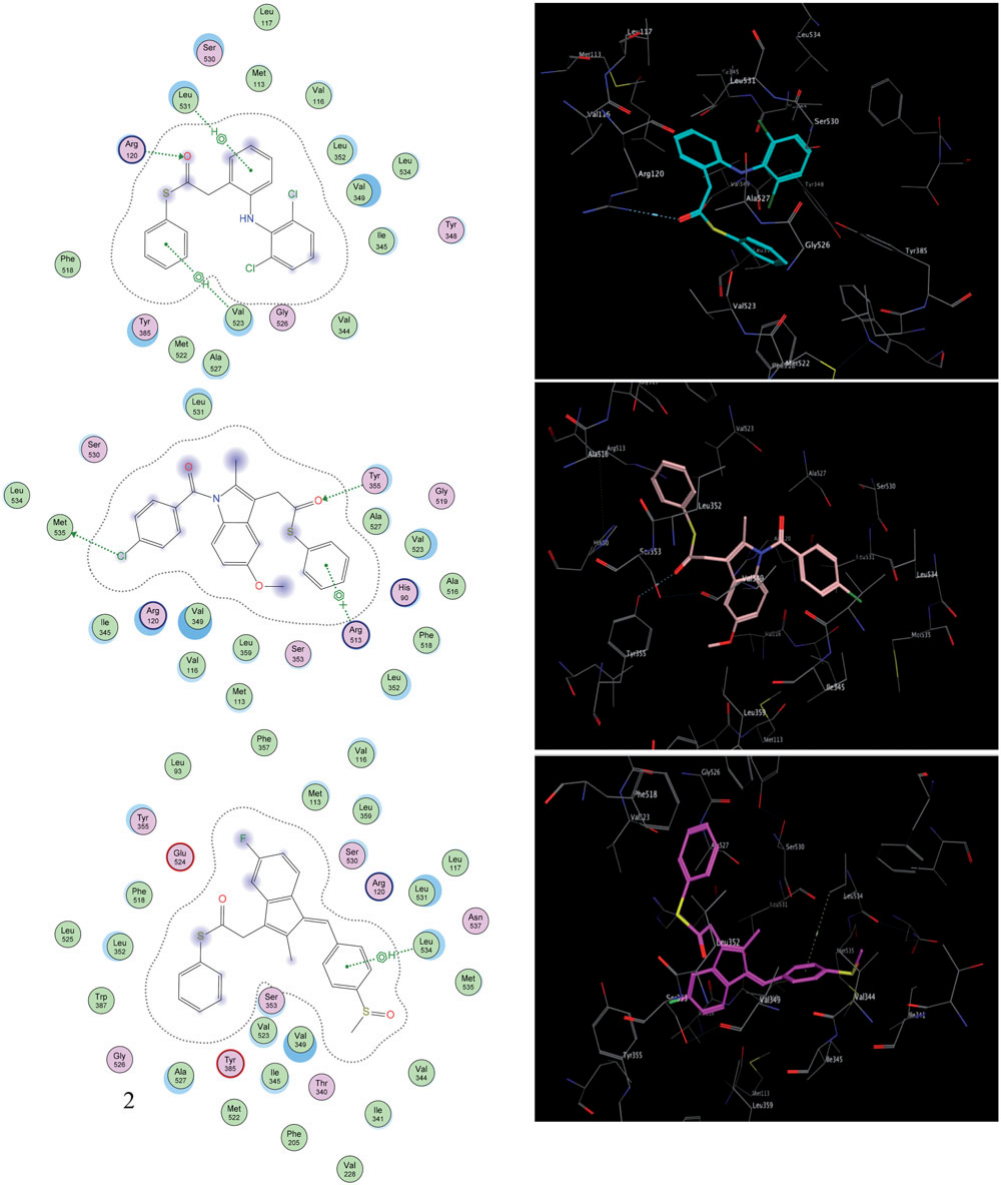
The 2D and 3D orientations of the docked compounds **6a** (upper panel), **7a** (middle panel), and **8a** (lower panel) in COX-2 active pocket (H bonds and hydrophobic interactions are shown as dashed green lines or arrows).

**Figure 3.**
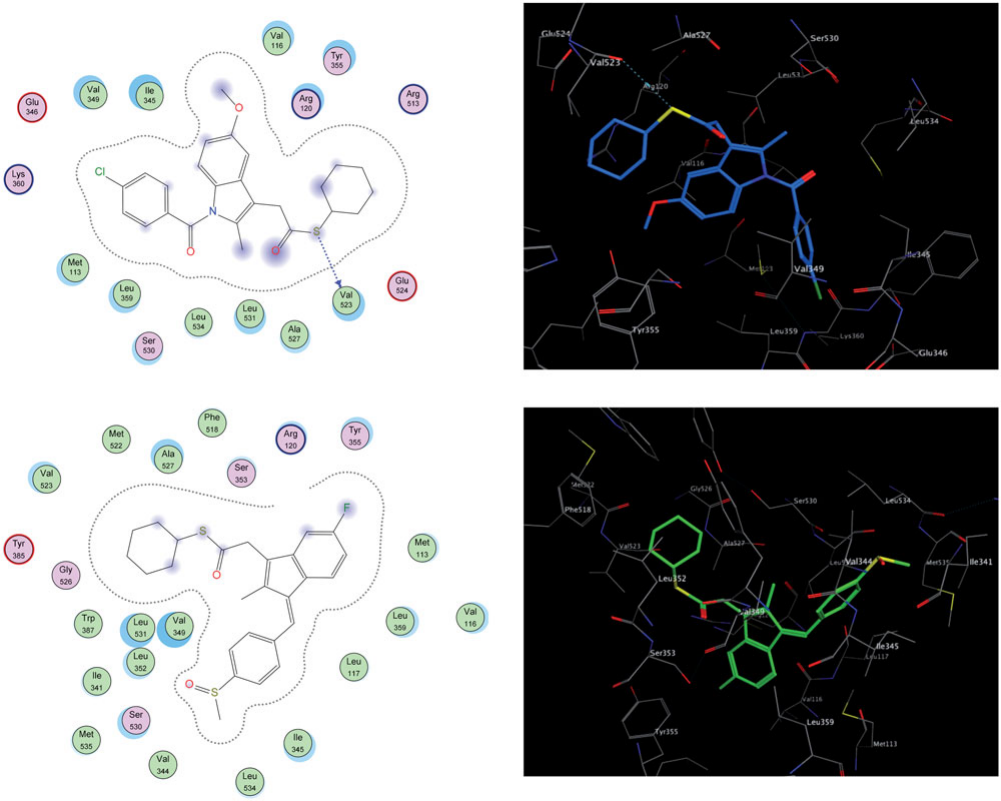
The 2D and 3D putative binding complexes of compound **7b** (upper panel) and compound **8b** (lower panel) within the binding pocket of COX-2 enzyme.

To highlight the inhibition selectivity of different core analogues towards the COX-2 enzyme, automated docking studies were carried out using the MOE 2008.10 program^54^. The scoring functions, hydrogen bonds and hydrophobic interactions formed with the surrounding amino acids are used to predict the binding modes, the energy of interaction and orientation of the docked compounds at the active sites of the COX-2 enzyme (Figures 2–3). The protein–ligand complex was constructed based on the X-ray structure of COX-2, with its bound inhibitor SC-558, which was available through the RCSB Protein Data Bank (PDB entry 1CX-2)^60^. The active site of the enzyme was defined to include residues within a 10.0-Å radius around any of the inhibitor atoms. This active pocket consisted of amino acid residues such as arginine (Arg^510^), histidine (His^90^), glutamine (Gln^192^) or tyrosine (Tyr^355^), arginine (Arg^120^), valine (Val^523^) and methionine (Met^535^), which play fundamental roles by forming H-bonds and hydrophobic interactions (Figures 2–3). In order to verify the reproducibility of the docking calculations, the cocrystallised ligand SC-558 was extracted from the complex and submitted for one-ligand run calculation. This reproduced 20 top scoring conformations falling within a root-mean-square deviation (rmsd) value between 0.4 Å and 2.0 Å, from the bound X-ray conformation for the COX-2 enzyme, suggesting that this method is valid enough to be used for docking studies of other compounds.

The present work is based on a comparative study to define the selectivity of most active COX-2 inhibitors, such as the thioester derivatives **6a**, **7a**, **7b**, **8a** and **8b** of well-known and well-established NSAIDs, namely diclofenac, indomethacin and sulindac, by exploring their docking and complementarity to the COX-2 binding site. Generally, the results of the docking study indicated that the thioesters based on indomethacin, sulindac and diclofenac scaffolds matched perfectly with the configuration of the T-shaped merged COX-2 binding site, which easily accommodated the wide bulk SC-558 inhibitor.

Compounds **8a** (IC_50_ = 0.20 µM), **7a** (IC_50_ = 0.22 µM) and **6a** (IC_50_ = 0.25 µM) were the most active analogues; they showed the highest recognition at the COX-2 binding site, which is consistent with the experimental results of the selectivity index obtained from the COX-2 assay (Table 2). Compound **8a** was shown to have a unique binding configuration (Figure 2, lower panel). The phenyl thioester of sulindac showed promising binding affinity and proper complementarity, because the E-conformer allows the crest-configuration to embed properly within the merged active site of the 5-flouroindenyl group, via proper hydrophobic interactions with the amino acids of the merged cleft active site. The thiocarbonyl function was impressively recognized with the polar amino acid Glu^524^. The two terminal phenyl groups are surrounded by hydrophobic amino acid analogs, where Leu^534^ showed an aromatic-aromatic interaction with the 4-methylsulfinylphenyl group. The terminal 4-methylsulfinyl fragment enhanced the strategic function that showed proper complementarity with the groove wall residues via both Val^344^ and Val^349^. According to the selectivity index and computational binding, the hydrogen-bonded compound **8a** was considered the most promising selective lead.

Moreover, compound **7a** was held by one hydrogen bond with Tyr^355^, via its carbonyl thioester, apart from the electrostatic interaction between the chloro-function and the mercapto moiety of the corresponding Met^535^ (Figure 2, middle panel). Aromatic recognition also was observed between the aromatic phenyl thioester and the side chain of Arg^513^.

Additionally, the docking studies of compound **6a** revealed outstanding interactions with one of the essential active-site Arg^120^ residues formed via proper hydrogen bonding (Figure 2, upper panel). The two aminophenyl and dichlorophenyl groups augment the aromatic-aromatic interaction with a series of seven hydrophobic amino acids, Leu^531^, Met^113^, Val^116^, Leu^352^, Val^349^ and Ile^345^, arranged in a continued chain, lining the wall of the cleft. However, because of the NH-amino group being embedded inwards and away from the surrounding residues, it does not interact with the active-site amino acids, owing to the bulkiness of the two phenyl substituents. The thiophenyl function protruded towards Val^523^, showing notable improvement in the net lipophilic stabilisation (Figure 2, upper panel).

In comparison to the aforementioned derivatives, compounds **7b** and **8b** showed moderately selective inhibition towards COX-2. Compound **7b** revealed distinct binding wherein the cyclohexyl group was merged with the side-pocket, and the thioester function was exposed to the surrounding binding residues for interaction with the conserved amino acids Val^523^ via proper hydrogen bonding. Additionally, methoxy oxygen was recognized by a single conventional hydrogen bond with the conserved Arg^120^ (Figure 3, upper panel).

Similarly, the cyclohexyl group of compound **8b** was merged with the side-pocket, and the ester function was exposed to the surrounding binding residues, to be oriented ahead of the polar amino acids, Ser^353^ and Arg^120^ (Figure 3, lower panel). Along the lining wall of the pocket, all the hydrophobic amino acids are oriented complementarily with the hydrophobic-facing groups indene, methylene, and the terminal phenyl. From another site, the following hydrophobic amino acids are stuffed properly and sandwiched between the cyclohexyl ring and the terminal phenyl group.

## Conclusions

A new series of thioesters based on NSAID scaffolds were synthesized and evaluated for their *in vitro* antitumor effects against a panel of four human tumour cell lines, namely HepG2, MCF-7, HCT-116 and Caco-2 using MTT assay. The thioesters **2b, 3b, 5b**, **7a**,**b**, and **8a** showed potent antitumor activity against HepG2 cell line, while thioesters **2a**, **3b, 6b**, **7a**,**b** and **8a** showed high sensitivity against MCF-7 cell line with IC_50_ values of 7.35–19.74 μM and 6.11–17.10 μM, respectively, compared with the reference drug, 5-FU, afatinib and celecoxib (IC_50_ = 7.91, 5.40, 25.60 μM and 5.43, 7.10, 31.28 μM, respectively). Additionally, thioesters **2a**, **3b, 6b**, **7a**,**b** and **8a** revealed the most antitumor activity against MCF-7 cell line, whereas HCT-116 has strong susceptibility to thioesters **2a**, **7a** and **8a** with IC_50_ values of 6.11–17.10 μM and 9.73–18.71 μM, respectively, compared with the reference drugs 5-FU, afatinib and celecoxib (IC_50_ = 5.43, 7.10, 31.28 μM and 6.85, 7.70, 42.74 μM, respectively). Additionally, the thioesters **2b**, **7a** and **8a** showed strong antitumor activities against HepG2 (IC_50_ ≅ 7.35–9.36 μM), MCF-7 (IC_50_ ≅ 8.62–11.86 μM), HCT-116 (IC_50_ ≅ 9.73–18.71 μM), and Caco-2 cell line (IC_50_ ≅ 15.44–21.73 μM). Thioesters **3b** and **7b** have broad-spectrum antitumour activity against HepG2 and MCF-7 cell lines (IC_50_ ≅ 10.52–17.10 μM), while thioesters **6a** showed broad-spectrum antitumor activity against the MCF-7 and Caco-2 cell lines (IC_50_ = 6.11 and 10.16 μM). According to their cytotoxicity activities, compounds **2b**, **3b**, **6a**, **7a**, **7b**, **8a** and **8b** were carefully chosen for mechanistic studies involving COX enzyme inhibition and kinase assays. *In vitro* COX-1/COX-2 enzyme inhibition assay results indicated that compounds **2b**, **3b**, **6a**, **7a**, **7b**, **8a** and **8b** selectively inhibited the COX-2 enzyme (IC_50_ = ∼0.20–0.69 μM), and SI values of (>72.5–250), compared to celecoxib (IC_50_ = 0.16 μM, COX-2 SI: > 312.5). Although all the tested compounds did not selectively inhibit the COX-1 enzyme (IC_50_ > 50 μM). On the other hand, EGFR, HER2, HER4 and cSrc kinase inhibition assays were evaluated at a concentration of 10 μM. The kinase inhibition assays indicated that compounds **2a**, **3b**, **6a**, **7a**, **7b** and **8a** showed no activity to negligible activity (% inhibition = ∼0–10%), comparable to an 81–100% inhibition of the reference drug Staurosporine at a concentration of 1 μM.

## Supplementary Material

Supplemental Material
