# Supplementary material for: Synthesis, antitumour activities and molecular docking of thiocarboxylic acid ester-based NSAID scaffolds: COX-2 inhibition and mechanistic studies
Source: J Enzyme Inhib Med Chem. 2018 May 28;33(1):989–98. doi: 10.1080/14756366.2018.1474878 (PMC6009944; doi:10.1080/14756366.2018.1474878)
Supplement: Supplemental Material [file IENZ_A_1474878_SM0339.zip › IENZ_1474878_Supplementary Material/S .I. Docking methodology.pdf]

Synthesis, antitumor activities, and molecular docking of thiocarboxylic acid ester-based NSAID scaffolds: COX-2 inhibition and mechanistic studies

Adel S. El-Azab[a,b]*, Alaa A.-M. Abdel-Aziz[a,c], Laila A. Abou-Zeid[d], Walaa M. El-Husseiny[d], Ahmad M. El_Morsy[b], Manal A. Al-Gendy[a] Magda A.-A. El-Sayed[d,e]

[a]*Department of Pharmaceutical Chemistry, College of Pharmacy, King Saud University, Riyadh 11451, Saudi Arabia*

[b]*Department of Organic Chemistry, Faculty of Pharmacy, Al-Azhar University, Cairo 11884, Egypt*

[c]*Department of Medicinal Chemistry, Faculty of Pharmacy, Mansoura University, Mansoura 35516, Egypt*

[a]*Department of Pharmaceutical Organic Chemistry, Faculty of Pharmacy, Mansoura University, Mansoura 35516, Egypt*

[e]*Department of pharmaceutical chemistry, Faculty of pharmacy, Horus university, New Damietta, Egypt*

Docking methodology

All modeling experiments were conducted with MOE programs running on PC computer [MOE 2008.10 of Chemical Computing Group. Inc]. (1)

Selection of protein crystal structure

Ligand-bound crystallographic structures of cyclooxygenase (COX-2) is available in the Protein Data Bank (http://www.rcsb.org/pdb/home/home.do). In this study, COX-2 complexed with SC-558 (1CX2) was evaluated and selected for docking. The errors of the protein were corrected by the structure preparation process in MOE. The first step in the generation of suitable protein structures is the assignment of hydrogen positions on the basis of default rules. All bound waters and cofactors contained in the PDB file have been removed. Finally, partial charges (the Gasteiger methodology) were calculated, and the active site of the ensemble has been defined as the collection of residues within 10.0 Å of the bound inhibitor and comprised the union of all ligands of the ensemble. All atoms located less than 10.0 Å from any ligand atom were considered.

Preparation of the ligand

The ligand coordinates were built using the builder tool of the MOE program. Next, the correct atom types (including hybridization states) and correct bond types were defined, hydrogen atoms were added, charges were assigned to each atom, and finally the structures were energy minimized (MMFF94x, gradient: 0.01). [(2)] The energies of ligand structures were previously minimized using the semi-empirical AM1 method [(3)] with MOE program [(1)]

Docking experiment

The docking experiment on COX-2 (1CX2) was carried out by superimposing the energy minimized ligand on SC558 in the PDB file 1CX2, after which SC-558 was deleted. The default Triangle Matcher placement method was used for docking. GBVI/WSA dG scoring function which estimates the free energy of binding of the ligand from a given pose was used to rank the final poses. The ligandeenzyme complex with lowest S_score was selected.

Refrences

.1 .MOE 200810 of Chemical Computing Group Inc Available from: http://wwwchemcompcom/press_releases/2008-11-04htm.

.2 Halgren TA. Merck molecular force field. I. Basis, form, scope, parameterization, and performance of MMFF94. Journal of computational chemistry. 1996;17(5- 6):490-519.

.3 Dewar MJ, Zoebisch EG, Healy EF, Stewart JJ. Development and use of quantum mechanical molecular models. 76. AM1: a new general purpose quantum mechanical molecular model. Journal of the American Chemical Society. 1985;10.9-3902:(13)7
