# Supplementary figures and images for: Synthesis, antitumour activities and molecular docking of thiocarboxylic acid ester-based NSAID scaffolds: COX-2 inhibition and mechanistic studies
Source: J Enzyme Inhib Med Chem. 2018 May 28;33(1):989–98. doi: 10.1080/14756366.2018.1474878 (PMC6009944; doi:10.1080/14756366.2018.1474878)

# Compound 1a

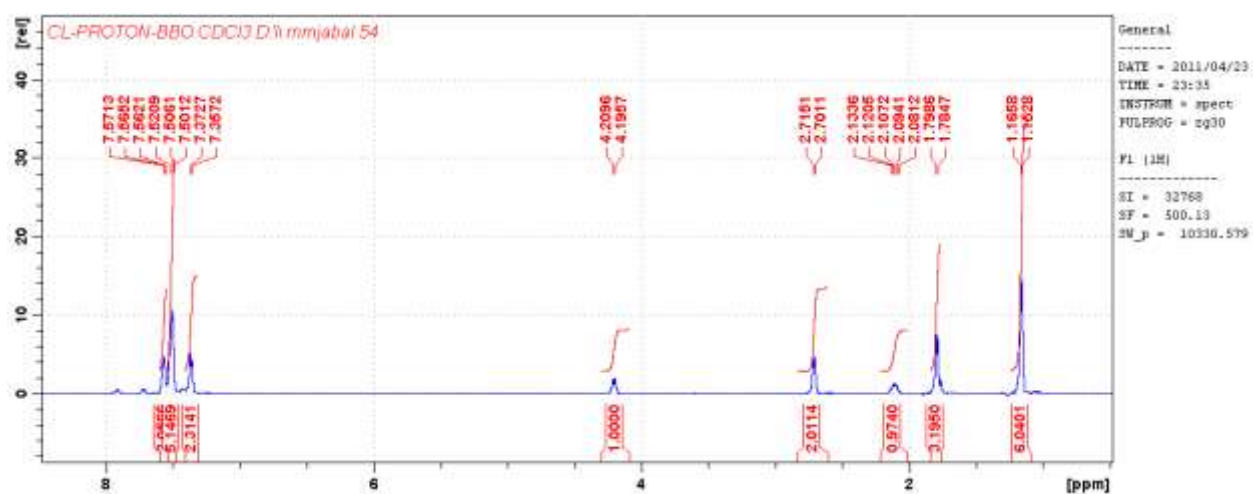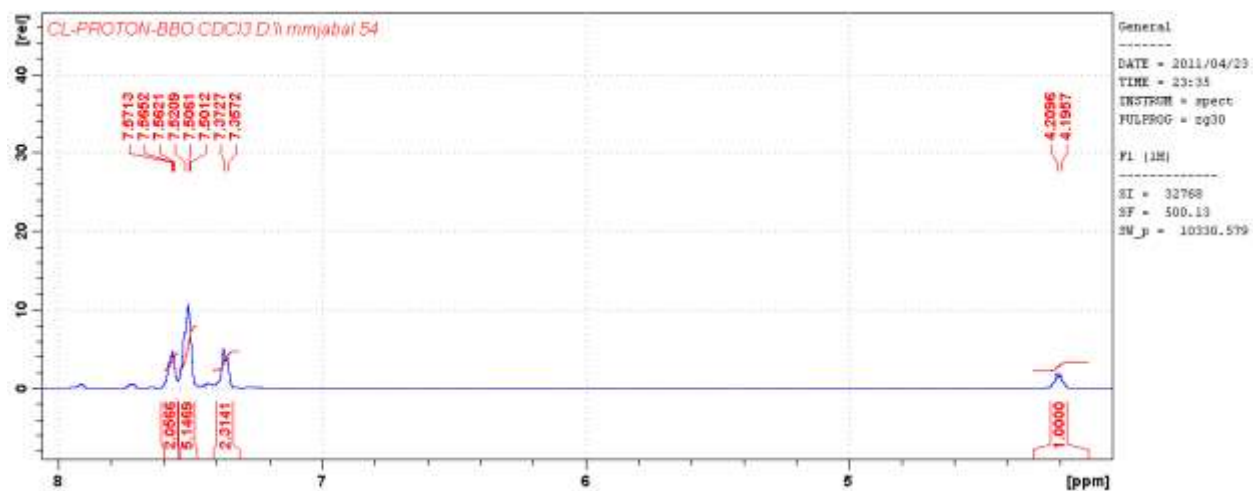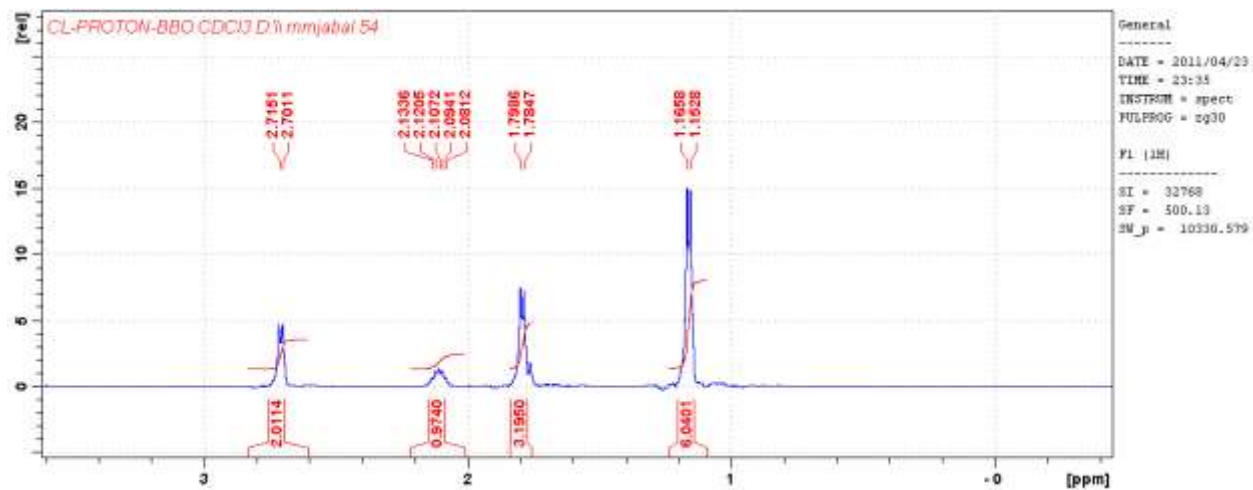

# Compound 1a

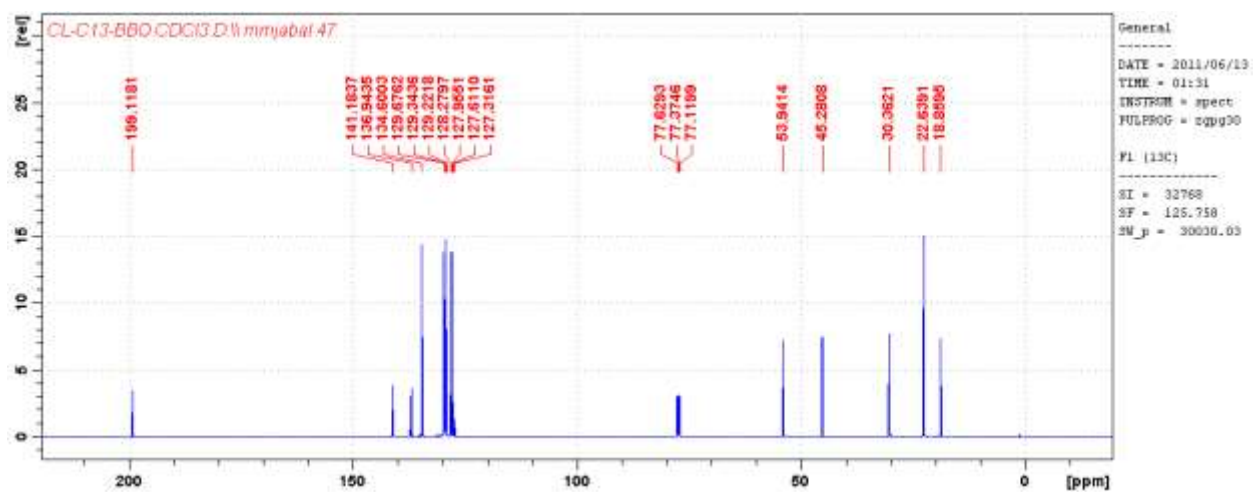

Supplement: Supplemental Material [file IENZ_A_1474878_SM0339.zip › IENZ_1474878_Supplementary Material/Compound 1a.pdf]

# Compound 1b

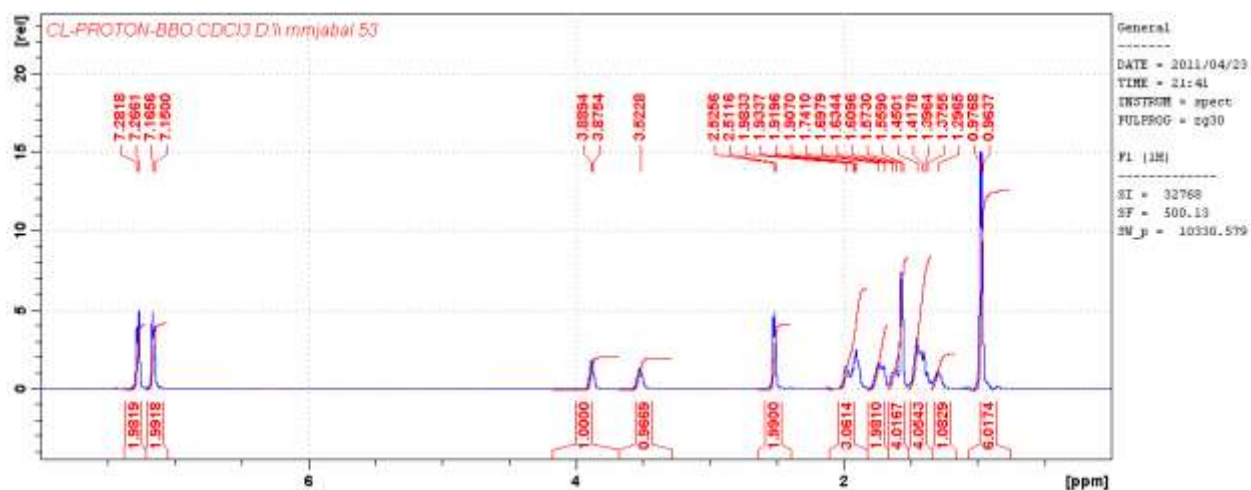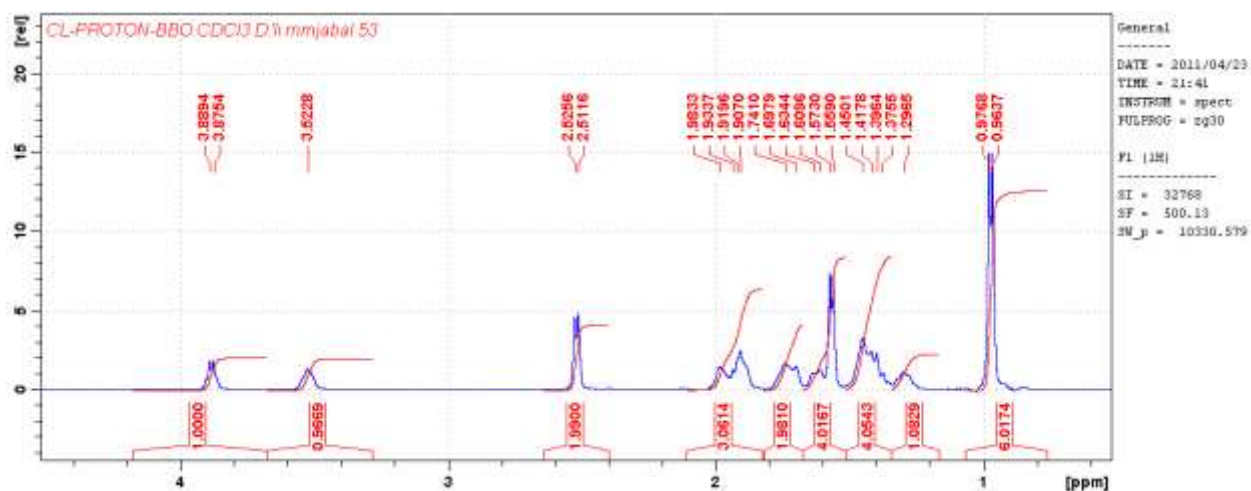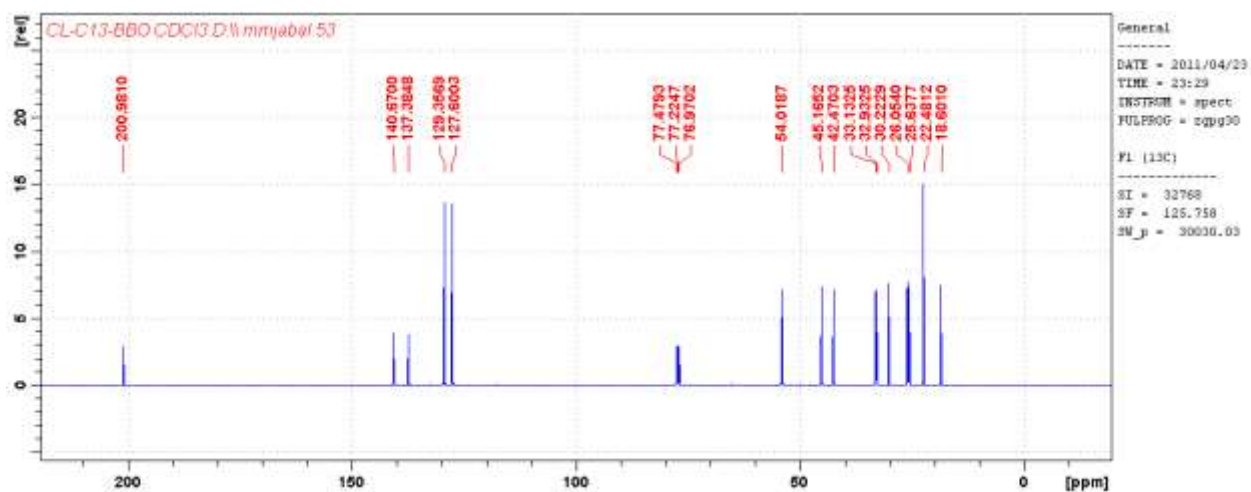

Supplement: Supplemental Material [file IENZ_A_1474878_SM0339.zip › IENZ_1474878_Supplementary Material/Compound 1b.pdf]

# Compound 2a

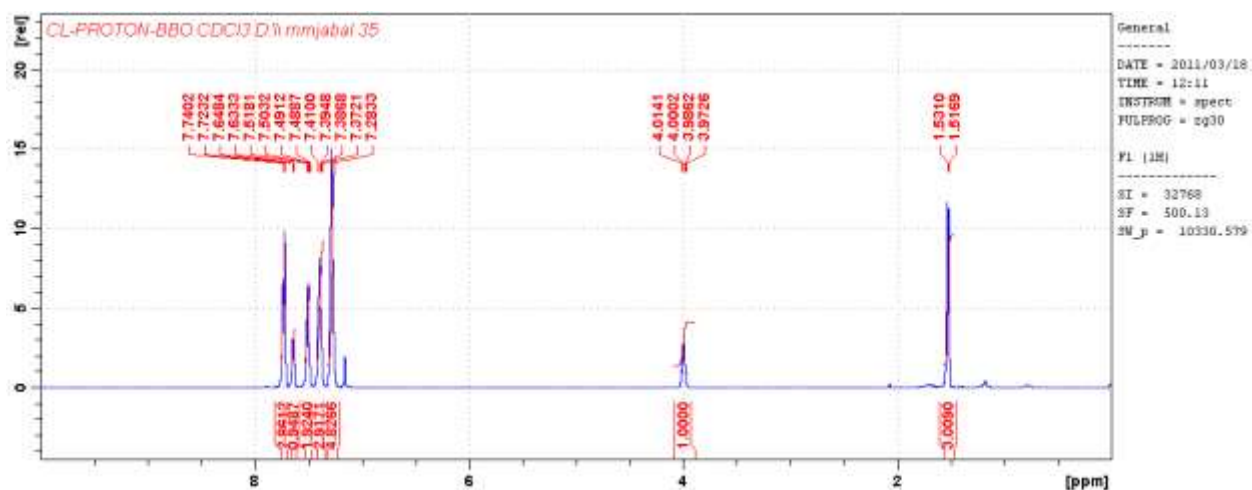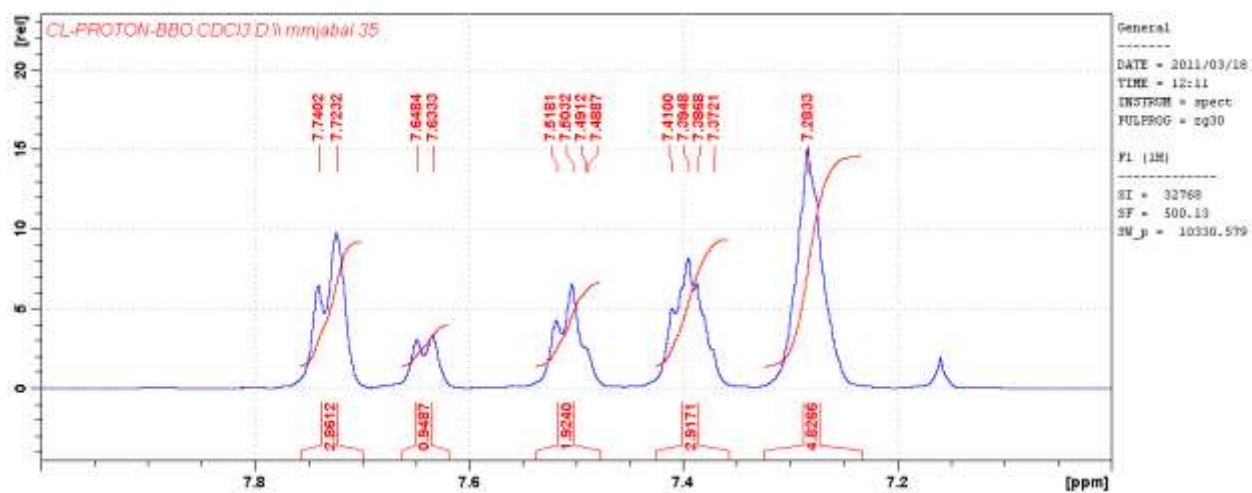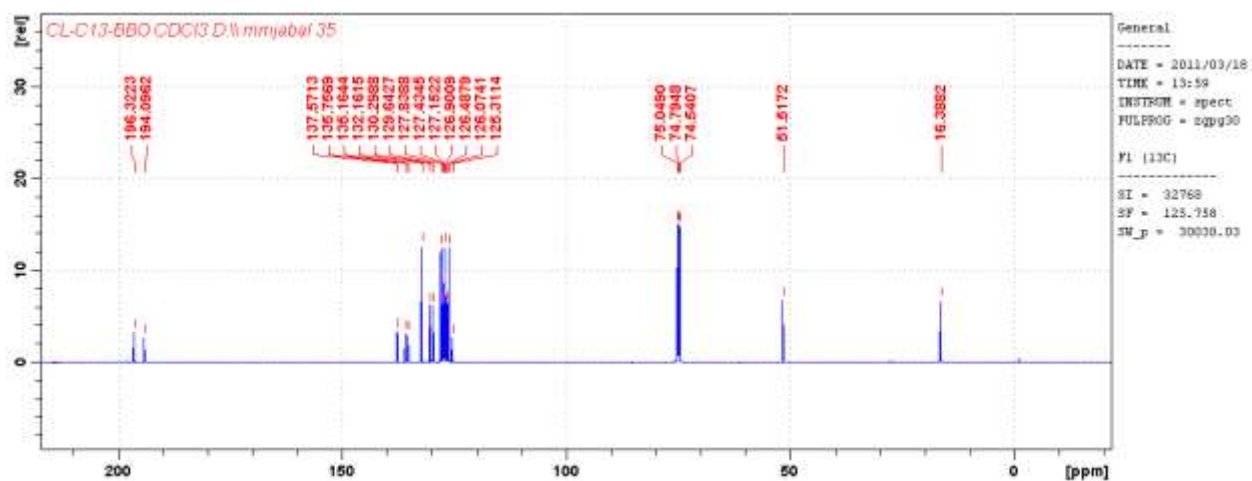

Supplement: Supplemental Material [file IENZ_A_1474878_SM0339.zip › IENZ_1474878_Supplementary Material/Compound 2a.pdf]

# Compound 2b

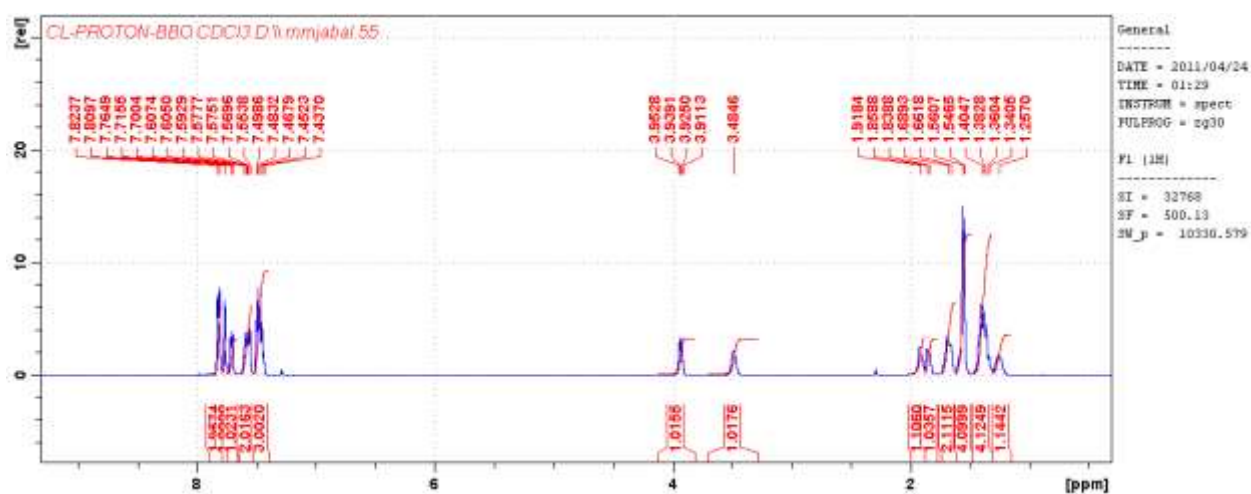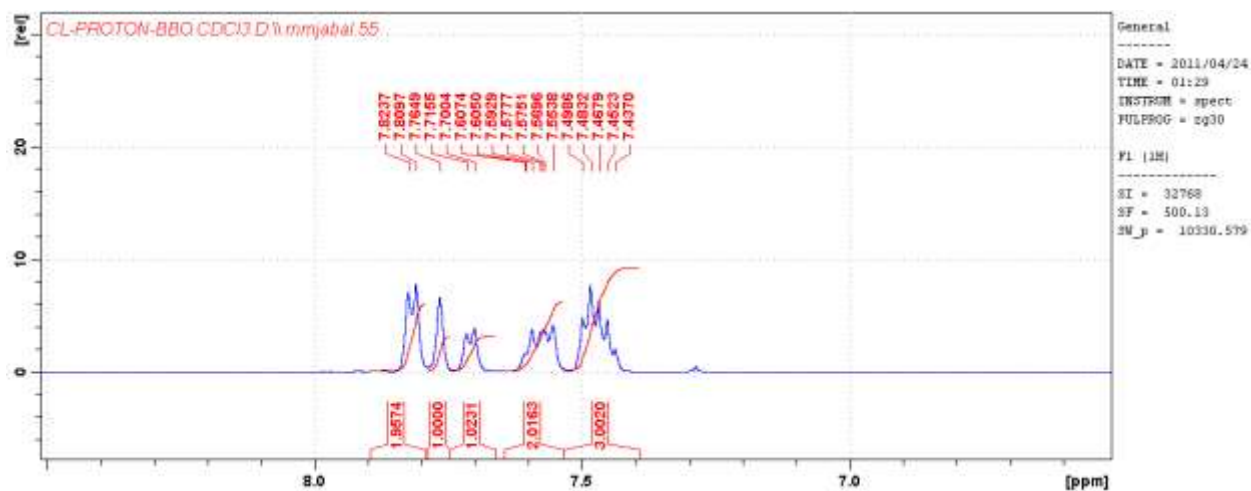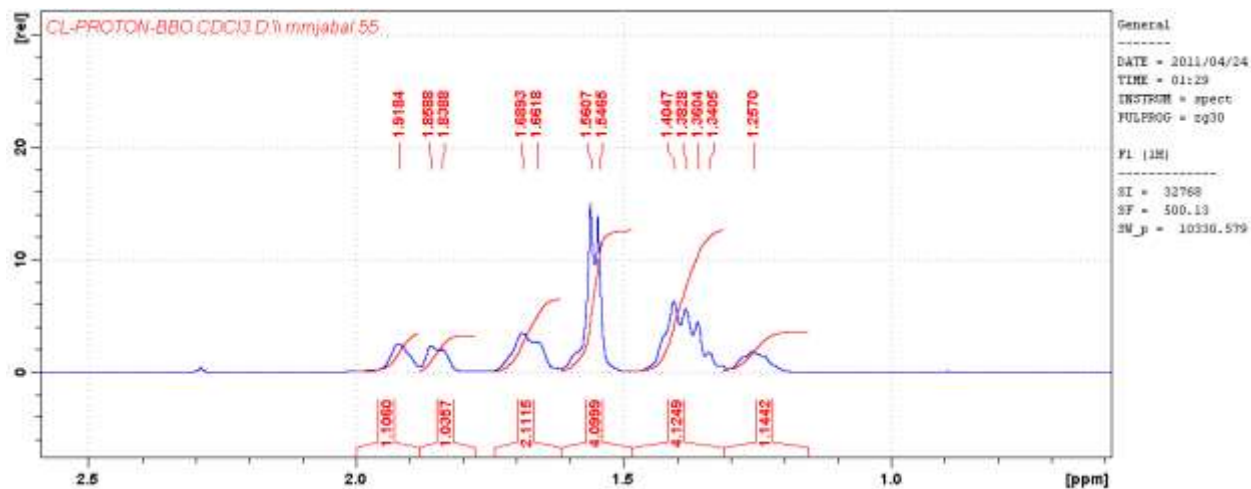

# Compound 2b

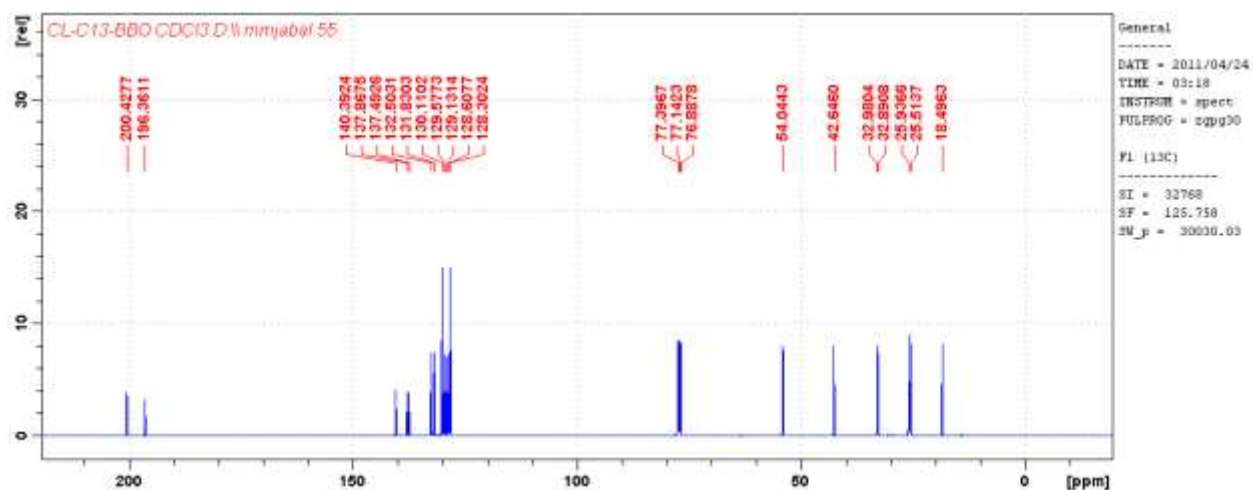

Supplement: Supplemental Material [file IENZ_A_1474878_SM0339.zip › IENZ_1474878_Supplementary Material/Compound 2b.pdf]

# Compound 3a

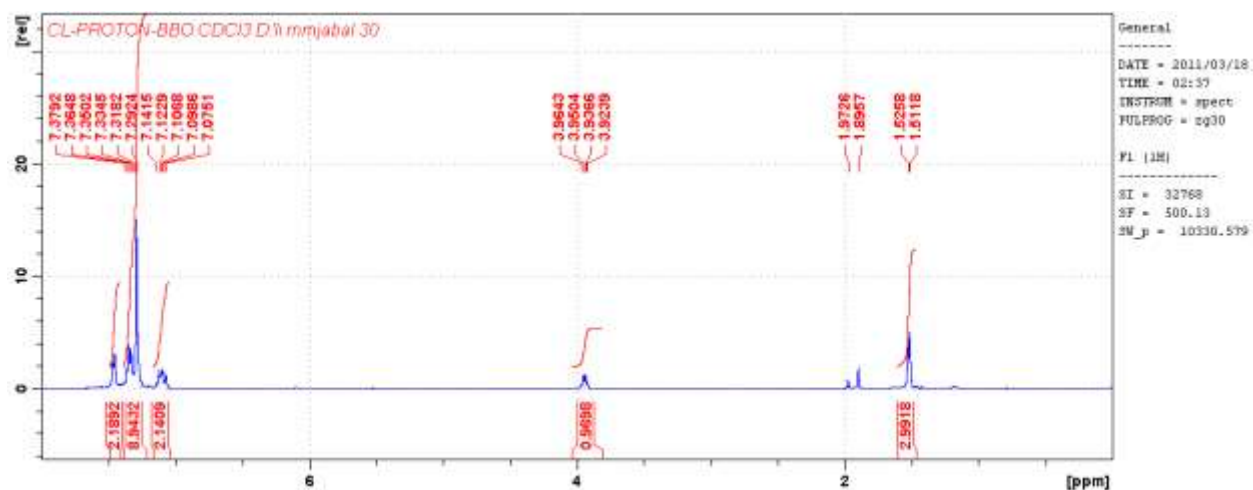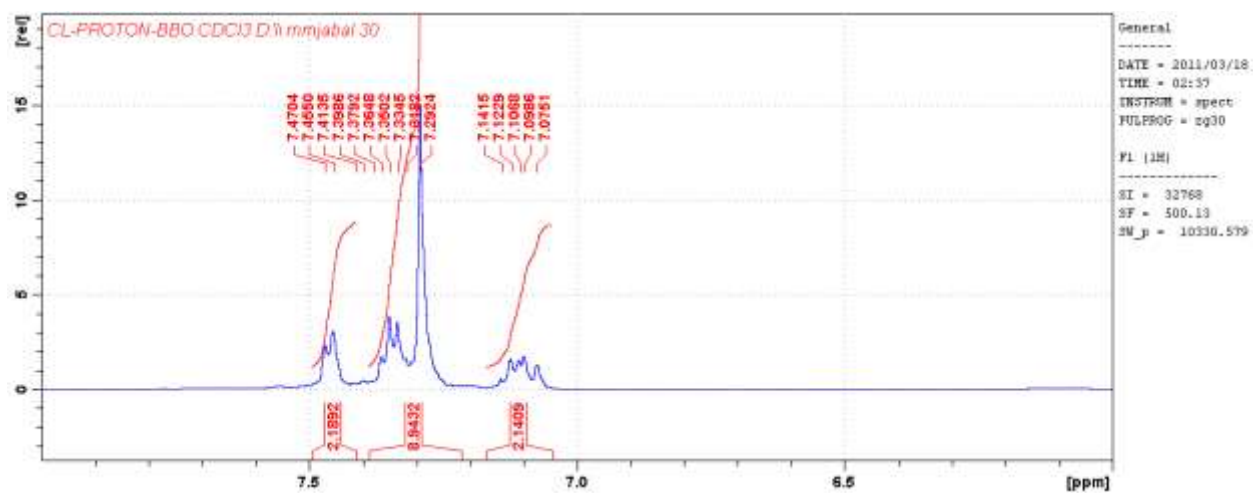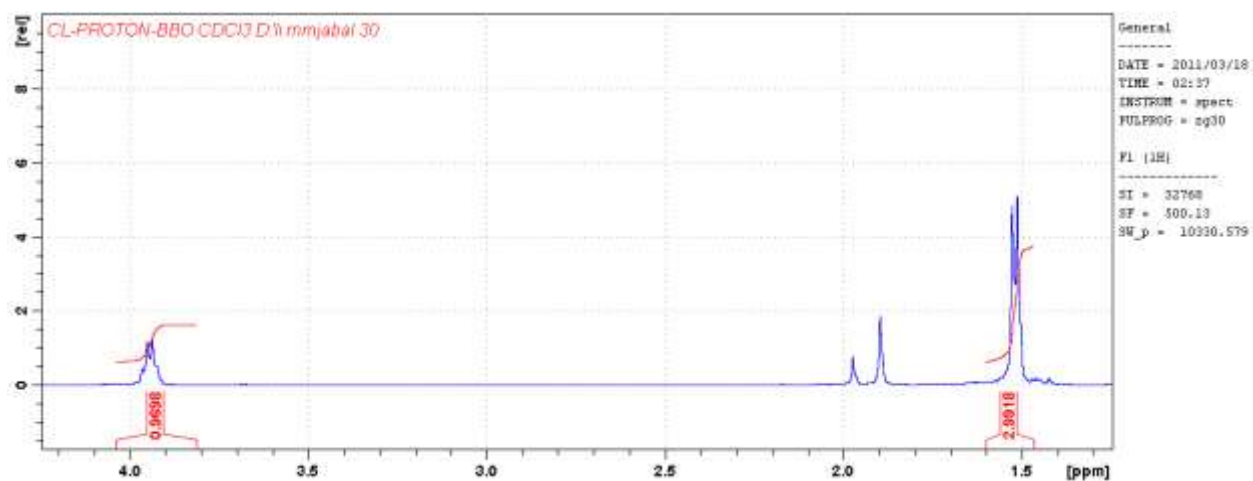

# Compound 3a

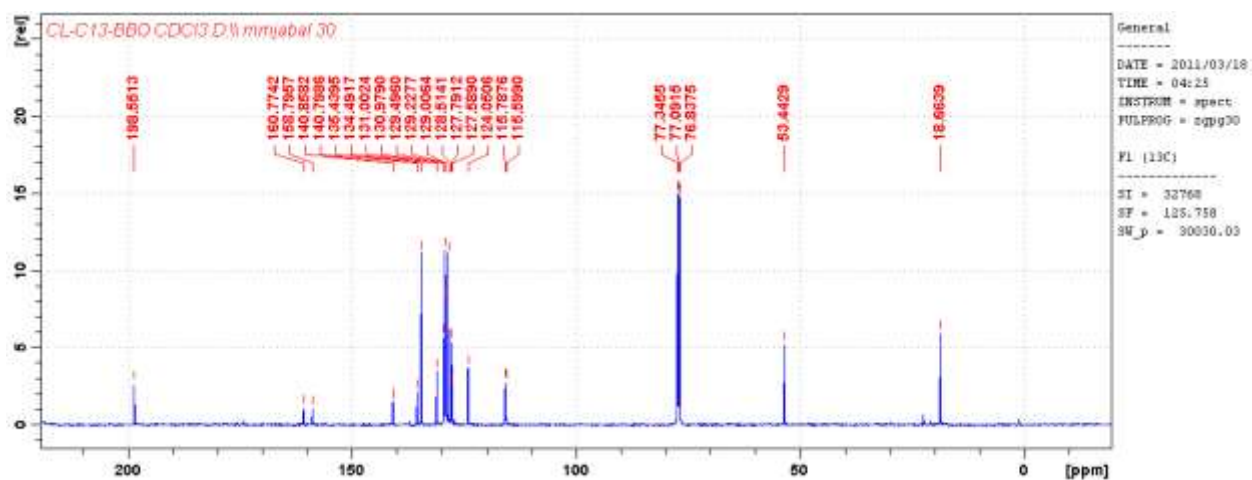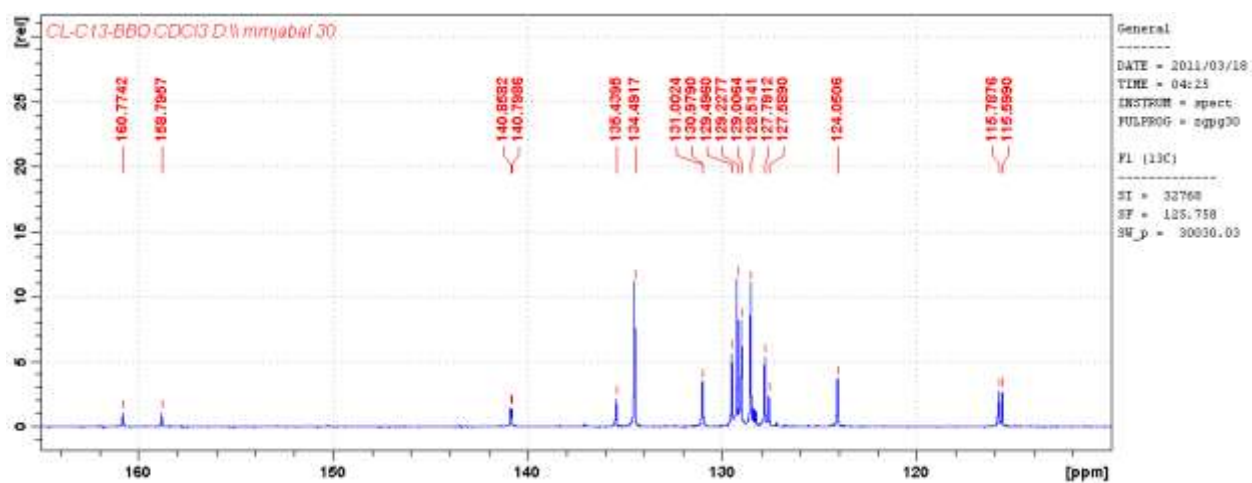

Supplement: Supplemental Material [file IENZ_A_1474878_SM0339.zip › IENZ_1474878_Supplementary Material/Compound 3a.pdf]

# Compound 3b

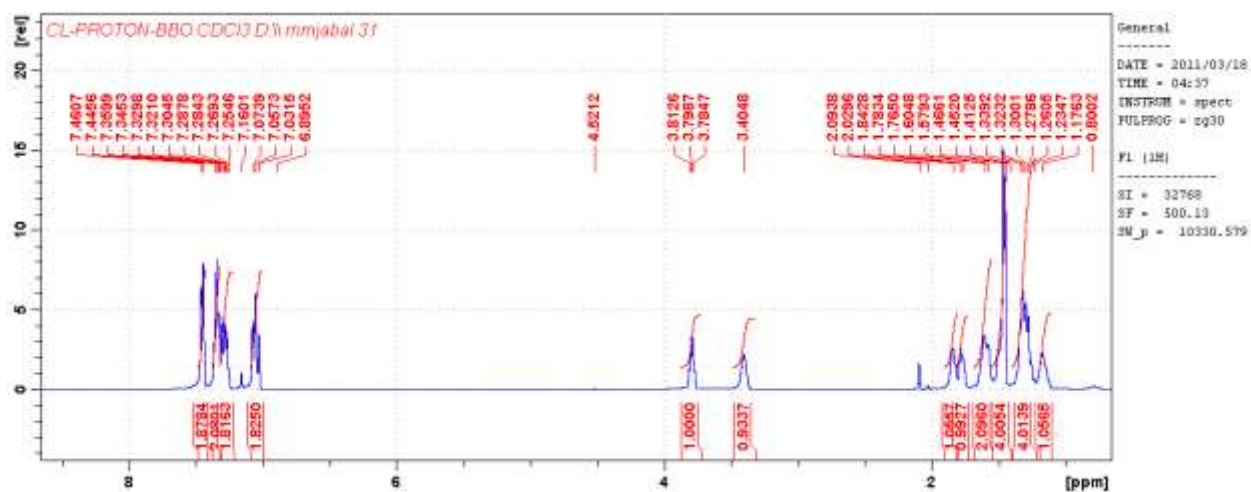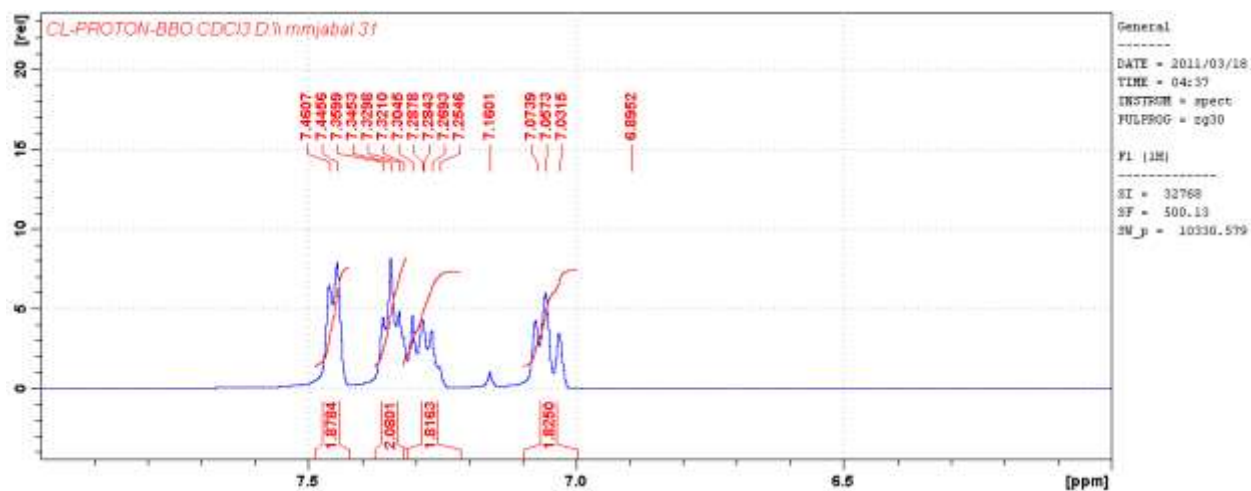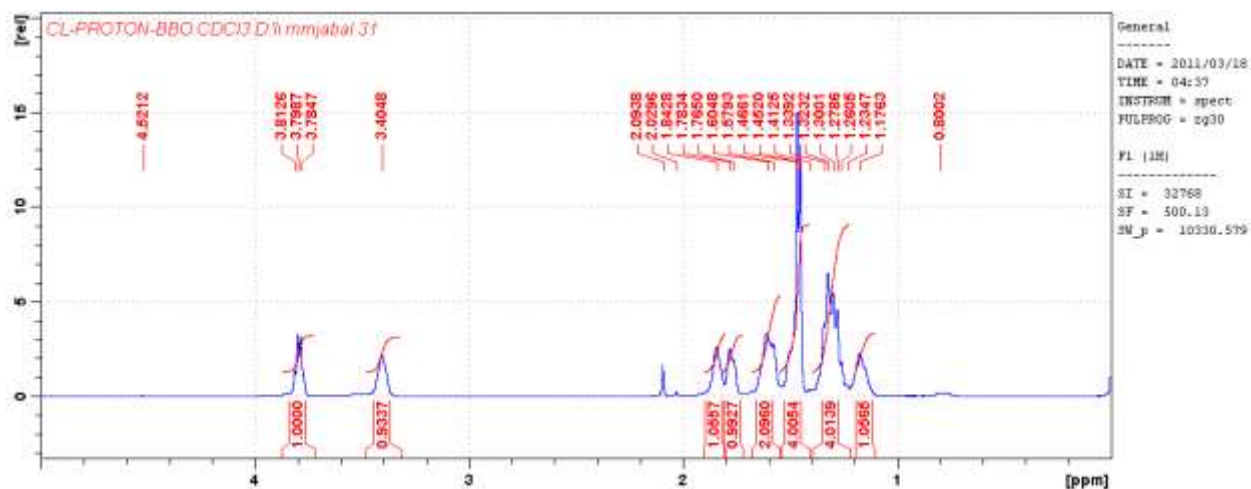

# Compound 3b

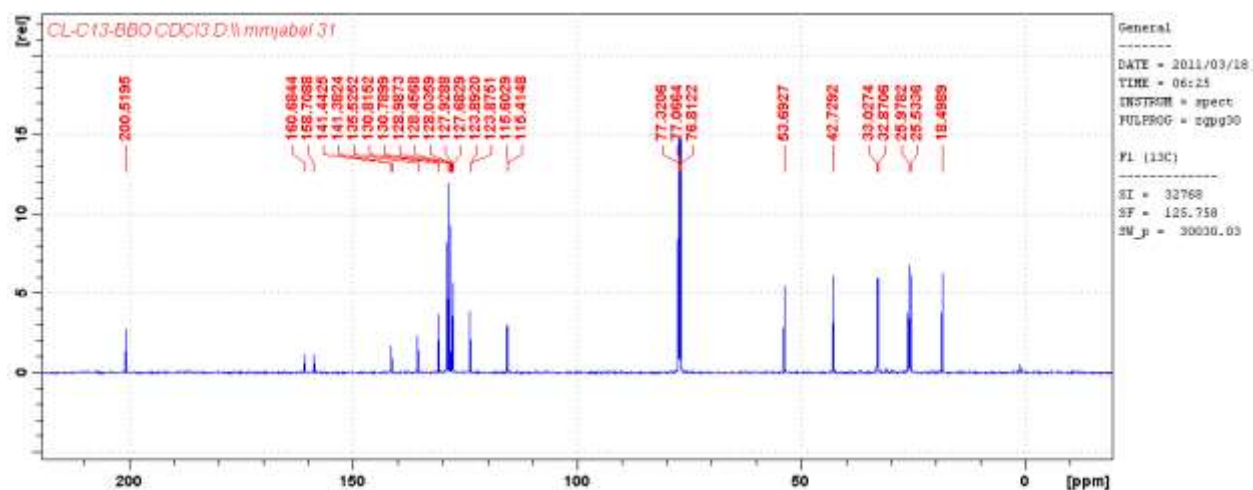

Supplement: Supplemental Material [file IENZ_A_1474878_SM0339.zip › IENZ_1474878_Supplementary Material/Compound 3b.pdf]

# Compound 5a

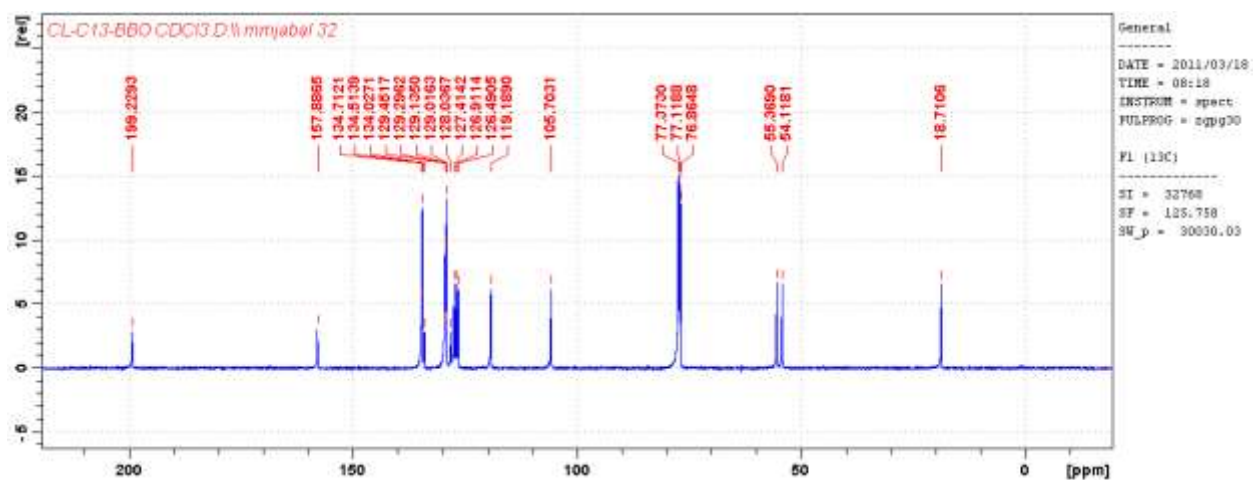

Supplement: Supplemental Material [file IENZ_A_1474878_SM0339.zip › IENZ_1474878_Supplementary Material/Compound 5a.pdf]

# Compound 5b

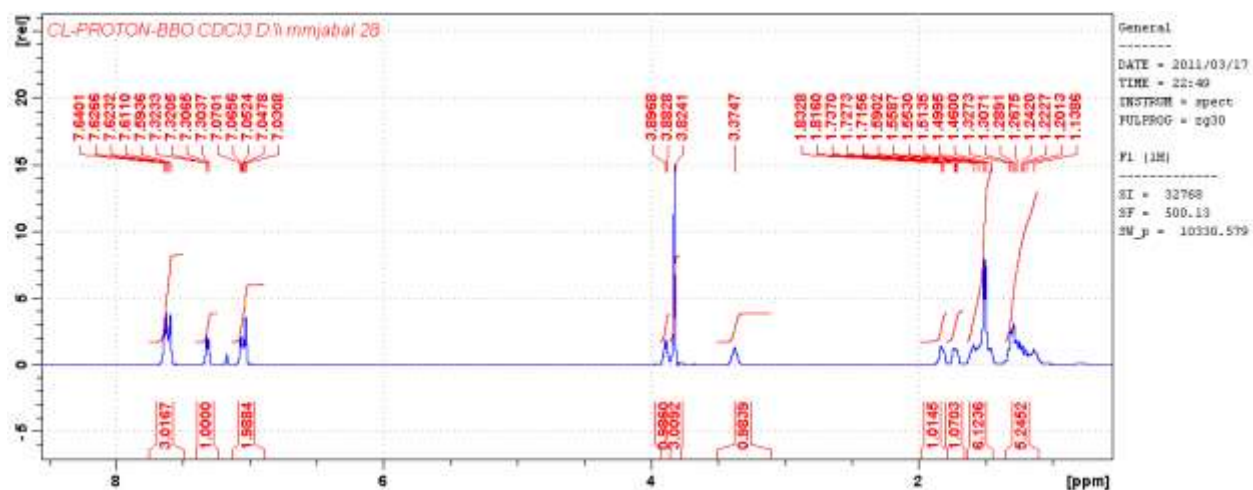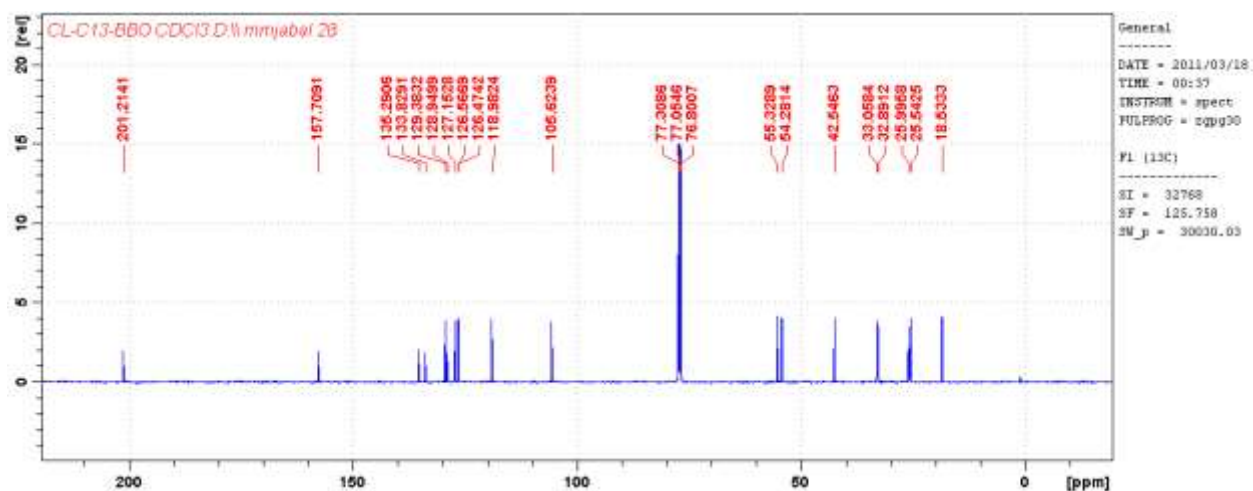

Supplement: Supplemental Material [file IENZ_A_1474878_SM0339.zip › IENZ_1474878_Supplementary Material/Compound 5b.pdf]

# Compound 7a

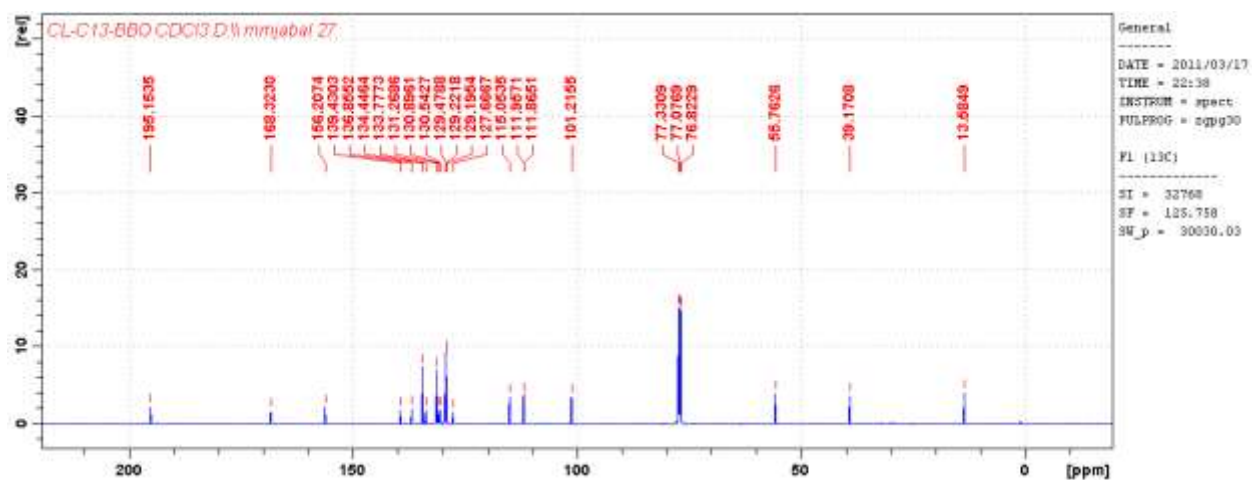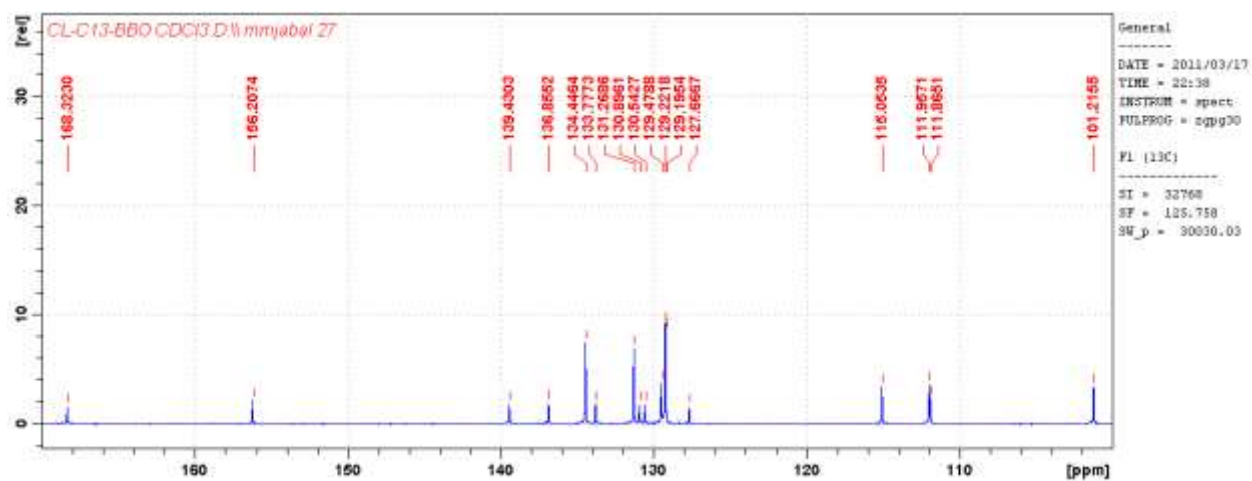

Supplement: Supplemental Material [file IENZ_A_1474878_SM0339.zip › IENZ_1474878_Supplementary Material/Compound 7a.pdf]

# Compound 7b

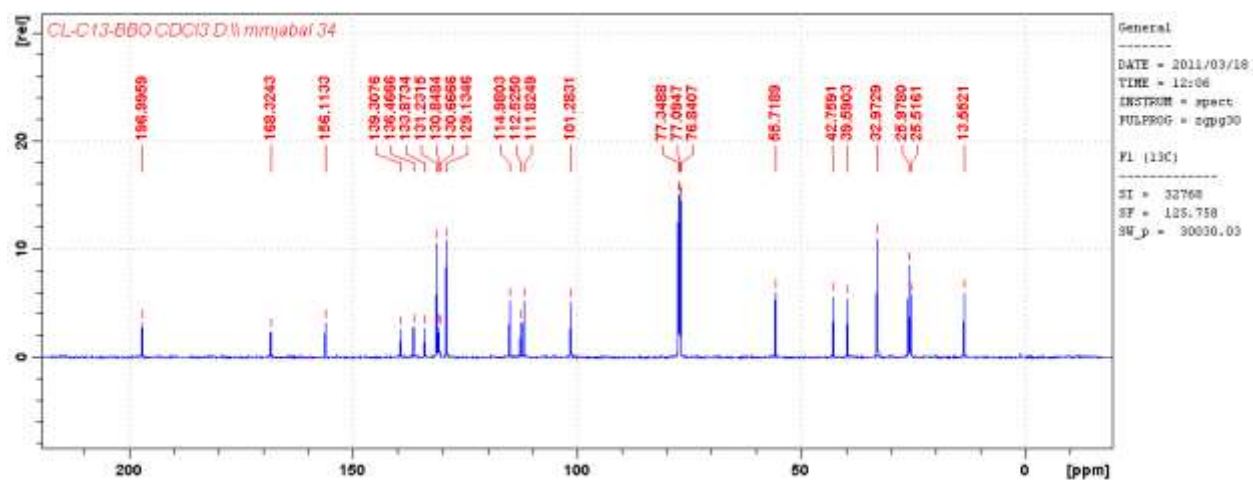

Supplement: Supplemental Material [file IENZ_A_1474878_SM0339.zip › IENZ_1474878_Supplementary Material/Compound 7b.pdf]

# Compound 8a

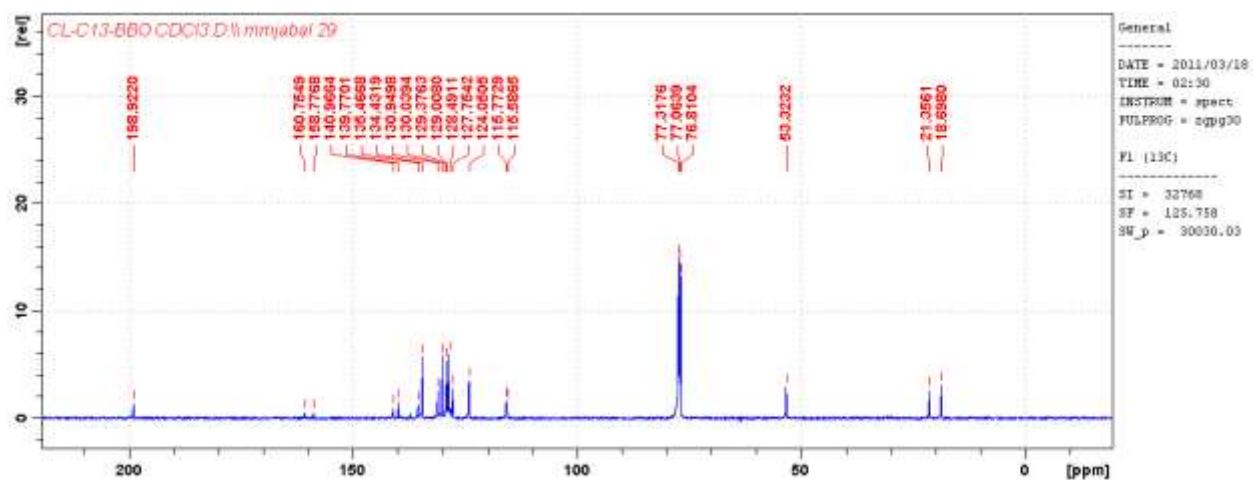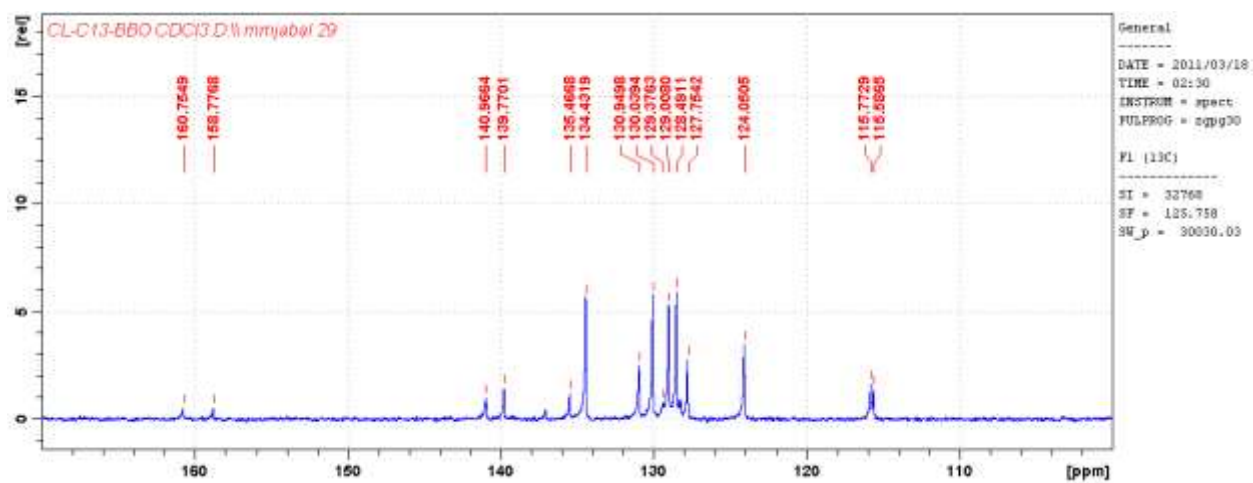

Supplement: Supplemental Material [file IENZ_A_1474878_SM0339.zip › IENZ_1474878_Supplementary Material/Compound 8a.pdf]

# Compound 8b

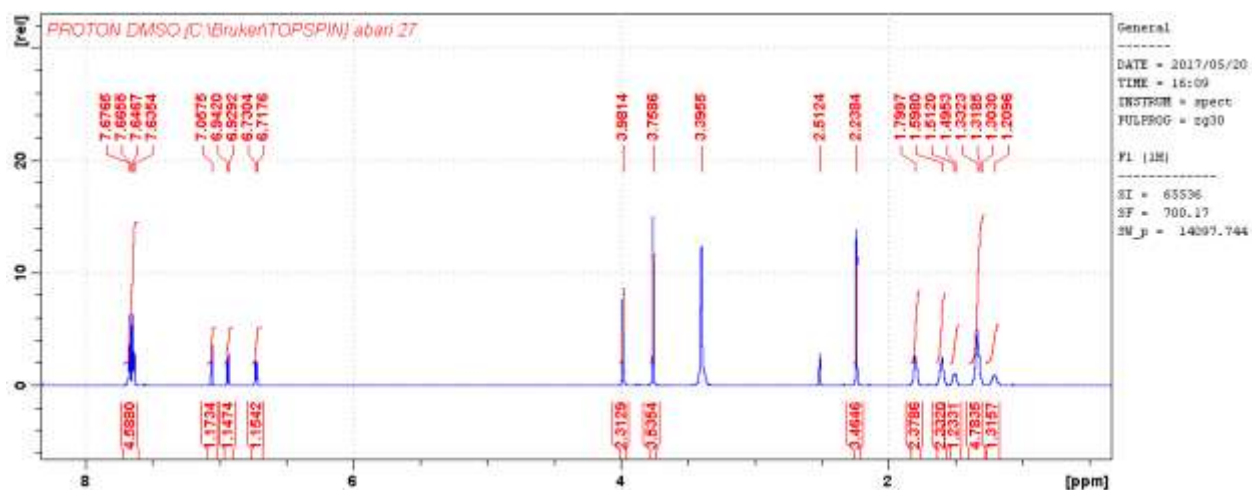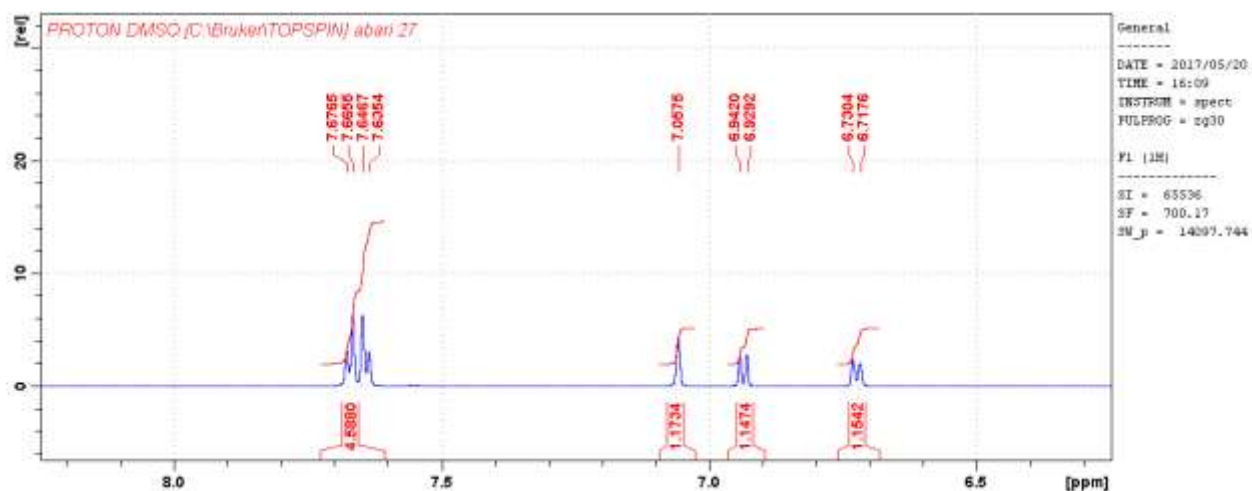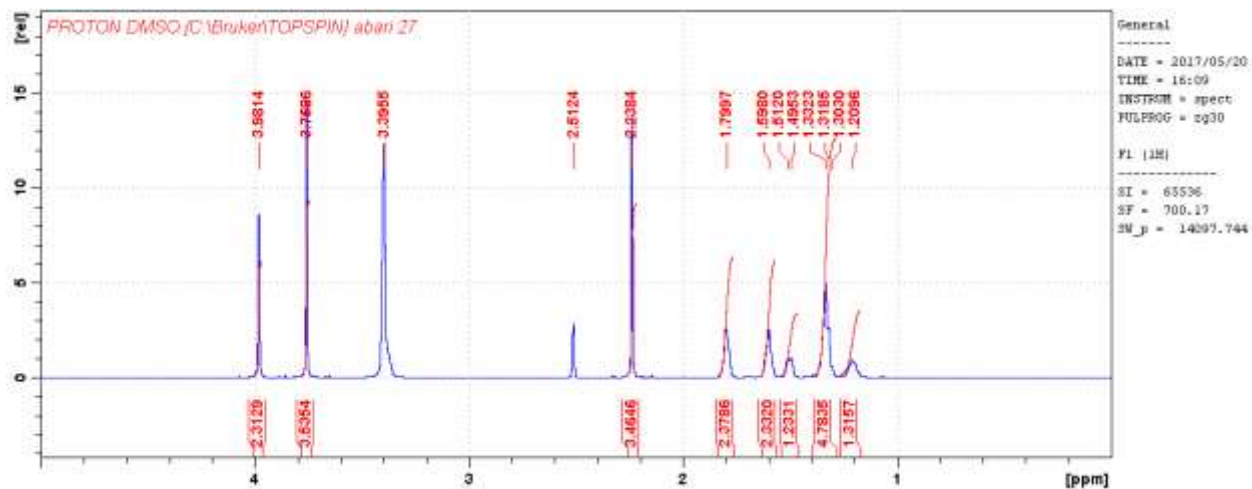

# Compound 8b

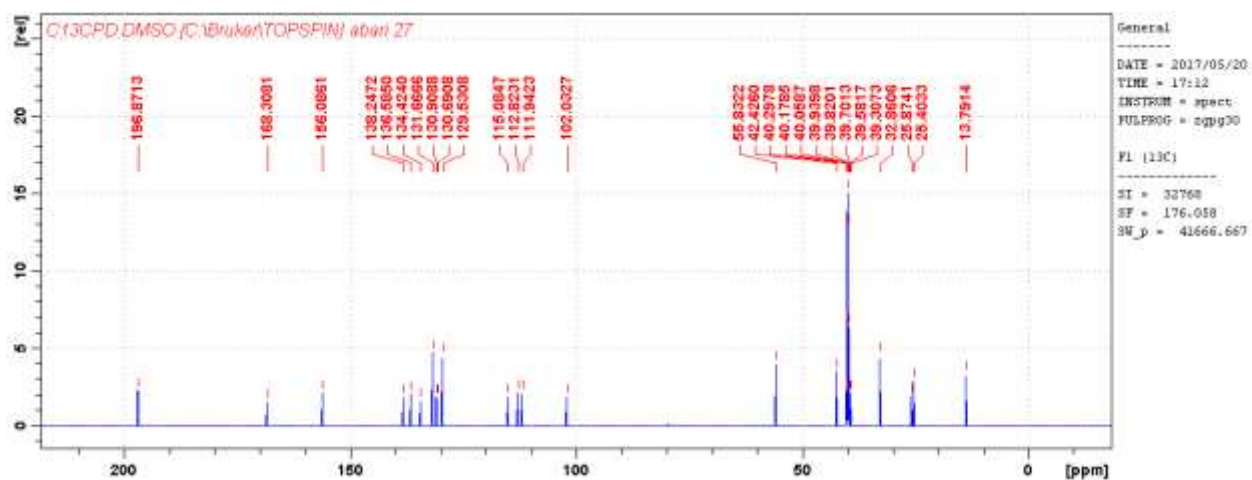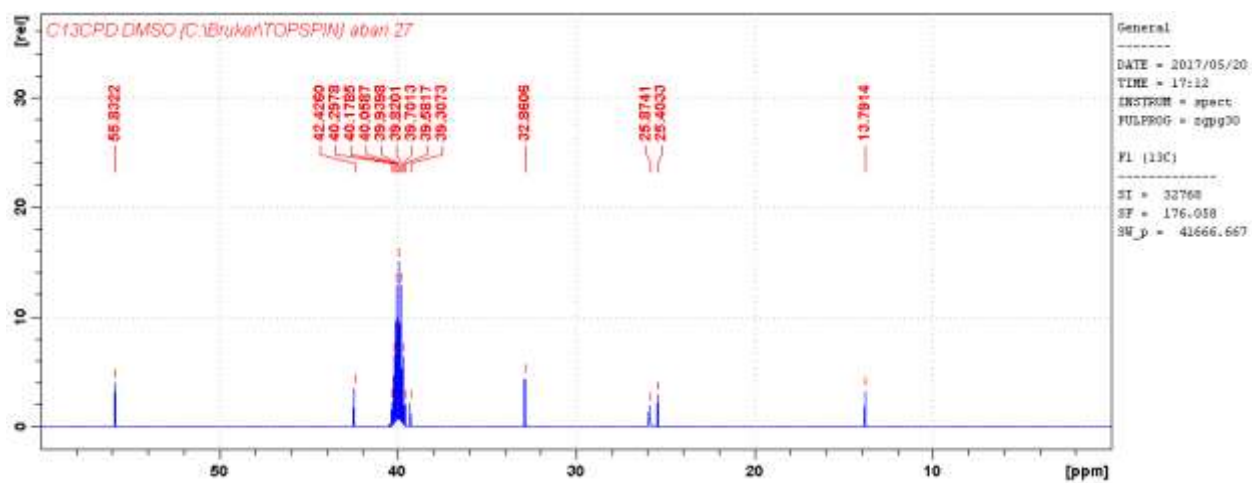

Supplement: Supplemental Material [file IENZ_A_1474878_SM0339.zip › IENZ_1474878_Supplementary Material/Compound 8b.pdf]
